# Pulmo–Cardio–Renal Continuum in Chronic Lung Diseases: A 3-Year Prospective Cohort Study

**DOI:** 10.3390/jcm14217631

**Published:** 2025-10-28

**Authors:** Lyazat Ibrayeva, Irina Bacheva, Assel Alina, Olga Klassen

**Affiliations:** Department of Internal Medicine, Karaganda Medical University, 100000 Karaganda, Kazakhstan; libraeva@qmu.kz (L.I.); bacheva@qmu.kz (I.B.); alina@qmu.kz (A.A.)

**Keywords:** systemic sclerosis–associated interstitial lung disease (SSc-ILD), chronic obstructive pulmonary disease (COPD), 6-min walk test (6MWT), exercise-induced desaturation, pulmonary hypertension (sPAP), NT-proBNP, MR-proANP, estimated glomerular filtration rate (eGFR), albumin-to-creatinine ratio (ACR), heart rate variability (HRV)

## Abstract

**Background/Objectives**: Systemic sclerosis-associated interstitial lung disease (SSc-ILD) and chronic obstructive pulmonary disease (COPD) are linked to multi-organ vulnerability involving the lungs, heart, and kidneys. This study aimed to compare the annual changes in pulmonary, cardiac, and renal parameters in patients with SSc-ILD and COPD across three consecutive years, using both individual biomarkers and integrated composite profiles. **Methods**: This observational longitudinal study included repeated assessments in 2023, 2024, and 2025. Functional, laboratory, and imaging parameters were collected: 6-min walk test (6MWT), SpO_2_ (pre-/post-exercise), spirometry/CT lung volumes, gas exchange (pO_2_/pCO_2_/lactate), echocardiography [left ventricular ejection fraction (LVEF), estimated systolic pulmonary artery pressure (sPAP)], cardiac biomarkers (NT-proBNP, MR-proANP, hsTnT), renal markers [eGFR, creatinine, albuminuria, albumin-to-creatinine ratio (ACR)], heart rate variability (HRV), and renal CT densitometry. All markers were standardized (z-scores, higher values = worse). Subprofiles were generated and aggregated into three integrated profiles (cardiac, renal, pulmonary). Within-group dynamics were analyzed using the Wilcoxon signed-rank test (year-to-year deltas), between-group comparisons with the Mann–Whitney U test, effect sizes via Cliff’s delta, and multiple testing correction with the Benjamini–Hochberg false discovery rate (FDR). **Results**: Exercise tolerance declined in both groups: by 2025, 6MWT distance decreased by −10 m in SSc-ILD (*p* = 0.006; q = 0.010) and −20 m in COPD (*p* = 0.002; q = 0.004); post-exercise SpO_2_ fell in both cohorts (both *p* < 0.001; q < 0.001). MR-proANP remained consistently higher in SSc-ILD across all years (*p* ≤ 0.005; q ≤ 0.028). sPAP increased in both groups, reaching higher values in COPD by 2025 (*p* = 0.007; q = 0.033). NT-proBNP and hsTnT increased over time, while eGFR declined, and ACR rose in both cohorts (both *p* < 0.001; q < 0.001). HRV (HF/total power) decreased by 2025. Composite profiles showed: in 2023, the cardiac profile was worse in SSc-ILD (δ ≈ 0.27; *p* = 0.011; q = 0.048), but differences diminished by 2025; the renal profile was initially worse in SSc-ILD but later shifted unfavorably in COPD; the pulmonary profile showed no consistent between-group differences. **Conclusions:** Over three years, patients with SSc-ILD and COPD exhibited concordant deterioration in pulmonary, cardiac, and renal function. Distinct leading markers emerged: desaturation during exercise and neurohormonal activation (MR-proANP) in SSc-ILD, versus reduced 6MWT and higher sPAP in COPD. These findings support the need for integrated monitoring of the cardio–pulmo–renal continuum. Limitations include the observational design, multiple comparisons, and absence of advanced repeated-measures modeling.

## 1. Introduction

Chronic lung diseases represent a substantial public health burden due to their progressive course and high risk of comorbid complications, particularly cardiovascular and renal involvement. Interstitial lung disease associated with systemic sclerosis (SSc-ILD) and chronic obstructive pulmonary disease (COPD) are traditionally studied from a pulmonary standpoint. However, growing evidence indicates that both disorders reflect a systemic pathophysiology encompassing multiple organ systems—the lungs, heart, and kidneys [1,2,3,4].

Damage to the alveolar–capillary barrier disrupts gas exchange and causes oxidative stress, endothelial dysfunction, and vascular injury in the lungs, heart, and kidneys [4,5,6,7]. Chronic hypoxemia contributes to pulmonary vascular remodeling and pulmonary hypertension, an independent predictor of mortality [7]. Fibrotic remodeling of the extracellular matrix further reduces lung compliance and exercise tolerance. Neurohormonal activation worsens the process: aldosterone causes fluid retention and reduces renal filtration, while galectin-3 from macrophages stimulates fibrosis [3]. Incomplete compensation of respiratory insufficiency sustains systemic fibrotic processes through reactive renal hyperfiltration. Together, these processes form the basis of the pulmo–cardio–renal continuum.

The pulmo–cardio–renal continuum expands the traditional cardio–renal syndrome by including the lungs as an initiating organ [4,8]. While the traditional model primarily focuses on the bidirectional relationship between the heart and kidneys, our approach integrates the lung as a potential initiating organ within this systemic axis. Chronic pulmonary injury can trigger a cascade of vascular, neurohormonal, and metabolic responses that secondarily affect cardiac and renal function. This broader perspective highlights the interdependence of the three organs and underscores that disturbances in pulmonary structure or function may propagate downstream dysfunction through shared mechanisms, including hypoxemia, endothelial injury, oxidative stress, and neurohormonal activation [9,10].

Recent studies confirm that both COPD and SSc-ILD exhibit systemic manifestations consistent with this continuum. In SSc, vascular injury and fibrosis extend beyond the lungs, frequently involving the myocardium and kidneys [11]. In COPD, chronic inflammation, endothelial dysfunction, and oxidative stress promote cardiac remodeling and renal impairment [9,12]. Meta-analyses have demonstrated that COPD is significantly associated with chronic kidney disease, and patients with combined pulmonary and renal impairment experience worse outcomes and higher mortality [10,13,14]. These findings underscore the need for integrative assessment of lung–heart–kidney interactions in chronic lung disease.

This study aimed to compare the dynamics of pulmonary, cardiac, and renal parameters in patients with SSc-ILD and COPD over three consecutive annual assessments, using both individual biomarkers and integrated composite profiles.

## 2. Materials and Methods

### 2.1. Study Design and Population

This was a prospective observational study in two patient cohorts (COPD and SSc-ILD) with three scheduled visits: 2023 (T0), 2024 (T1), and 2025 (T2). Recruitment was carried out at the Regional Clinical Hospital between January and December 2023.

**Figure 1:**
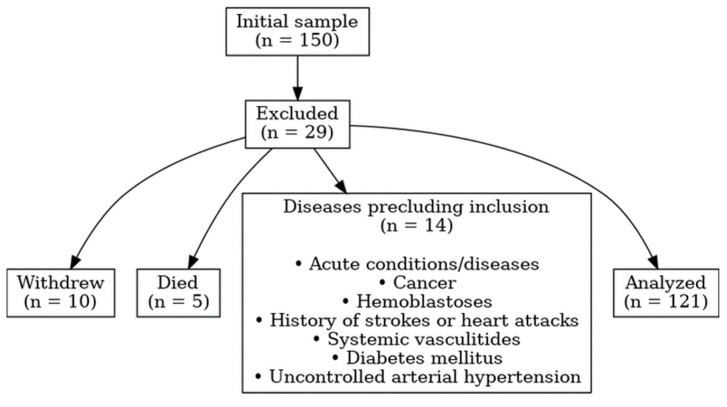
Flow diagram of patient selection.

A total of 150 patients were initially enrolled. By the final visit, 121 patients had completed the study: 62 with COPD and 59 with SSc-ILD (Figure 1). Only patients who completed all three visits were included in the analysis.

Inclusion criteria: Confirmed COPD (GOLD, spirometry with FEV_1_/FVC < 0.7); confirmed SSc-ILD (ACR/EULAR criteria with HRCT evidence of interstitial lung disease); smoking history > 10 pack-years for all COPD patients. Exclusion criteria: age < 25 or >65 years; chronic heart failure with reduced or mildly reduced ejection fraction; pregnancy; baseline eGFR < 60 mL/min/1.73 m^2^; acute infections or disease exacerbations; solid or hematological malignancy; prior stroke or myocardial infarction; systemic vasculitis; diabetes mellitus; uncontrolled arterial hypertension.

Sample size and statistical power. Sample size estimation was performed using R version 4.3.2 (R Foundation for Statistical Computing, Vienna, Austria) (package pwr) to ensure adequate statistical power for between-group comparisons. According to pwr.anova.test (k = 4, f = 0.25, α = 0.05, power = 0.75), the minimum required sample size was *n* = 37. A total of 150 participants were initially enrolled to account for attrition during the three-year follow-up; 121 completed the study. This sample size was considered sufficient for exploratory analyses, particularly given the repeated measurements and the use of composite indices that increase internal robustness.

Ethics approval: The study was approved by the local ethics committee (approval code: 2/11-10-2022). All participants provided written informed consent. Patient data were anonymized and assigned unique identifiers.

### 2.2. Baseline Characteristics

In the COPD cohort (*n* = 62), men accounted for 86.3% (*n* = 50) and women 13.7% (*n* = 8). The median age was 56 years [IQR 52.0–60.0]. All participants had a smoking history (100%, *n* = 58).

In the SSc-ILD cohort (*n* = 59), women predominated (77.8%, *n* = 42), while men accounted for 22.2% (*n* = 12). The median age was 53 years [IQR 49.0–57.0]. Only a small proportion reported a smoking history (5.5%, *n* = 3).

### 2.3. Clinical Assessment

6-Min Walk Test (6MWT). Conducted in a 30-m corridor according to ATS guidelines with standardized instructions. Distance walked, heart rate (HR), blood pressure (BP), and SpO_2_ were measured before and after the test, along with Borg scale ratings.

Arterial blood gases. Arterial blood samples were collected in a seated position and analyzed using the epoc Blood Analysis System (Siemens Healthineers, Erlangen, Germany), measuring pO_2_, pCO_2_, pH, lactate, and sodium. The system requires ~90 µL of whole blood and delivers results in <1 min.

Capnography. Exhaled CO_2_ concentration was measured using a portable Capnostream 35 capnograph/pulse oximeter (Covidien LLC, Jerusalem, Israel). Each recording followed 5 min of rest, was repeated three times, and the median value was used for analysis. A single-use O_2_/CO_2_ Nasal FilterLine cannula (Oridion Medical 1987 Ltd., Jerusalem, Israel) was applied for each patient.

Echocardiography. Performed using a VIVID IQ ultrasound system (GE Healthcare, Wuxi, China) with a 1.3–4.0 MHz 3Sc-RS phased-array transducer, following ASE/EACVI recommendations. Early (E) and atrial (A) diastolic velocities, E-wave deceleration time, and tissue Doppler e′ (average of septal and lateral mitral annulus) were measured. The E/e′ ratio was calculated to estimate LV filling pressure. Left atrial volume was determined using the biplane Simpson method.

Heart rate variability (HRV). HRV was assessed using a Polar H10 chest-strap ECG sensor (Polar Electro, Kempele, Finland), validated in research settings for high-precision R-R interval detection. A 5-min recording was obtained in a seated resting position. Artifacts and ectopic beats were excluded. Time-domain (e.g., SDNN, RMSSD) and frequency-domain (LF, HF, TP) indices were computed according to Task Force standards (1996) [15].

Spirometry. Forced vital capacity (FVC) and forced expiratory volume in one second (FEV_1_) were measured with a BTL-08 Spiro Pro device (BTL Industries Ltd., Ashford, UK), in line with European Respiratory Society guidelines.

Computed tomography (CT). High-resolution CT was performed using a 160-slice Aquilion PRIME SP scanner (Canon Medical Systems, Ottawa, Japan; B70f kernel, 1 mm slices, 120 kV, automatic mA modulation). Quantitative densitometry was analyzed with VIDAR DICOM Viewer v3.0 (VIDAR Systems, Herndon, VA, USA). Readers were blinded to clinical and laboratory data.

Laboratory markers. All blood samples were processed within 30 min after collection, aliquoted, and stored at −80 °C until analysis. Each sample underwent no more than one freeze–thaw cycle, and all biomarker measurements were performed within three months according to the manufacturers’ protocols.

hs-TnT: Cloud-Clone Corp. (Katy, TX, USA).

MR-proANP: Cusabio (Houston, TX, USA).

NT-proBNP and Galectin-3: Measured on the Alinity system (Abbott, Abbott Park, IL, USA) in the accredited OLYMP laboratory (Karaganda, Kazakhstan; ISO 15189:2015 [16]).

Endothelin-1: competitive ELISA (Cloud-Clone Corp., Houston, TX, USA; kit CEA482Hu; LoD < 2.7 pg/mL), performed at the Research Laboratory of Karaganda Medical University using an EVOLIS automated analyzer (Bio-Rad, Hercules, CA, USA).

Serum creatinine and albumin-to-creatinine ratio (ACR): Photometric method; eGFR calculated by the CKD-EPI (2021) equation) [17].

### 2.4. Statistical Analysis

The distribution of each variable was assessed using the Shapiro–Wilk test. Since none of the variables followed a normal distribution (*p* < 0.05), non-parametric methods were applied. Paired observations from 2023 to 2024 within each cohort were compared using the two-sided Wilcoxon signed-rank test. Between-group differences were analyzed with the Mann–Whitney U test, with p-values adjusted for multiple comparisons using the Benjamini–Hochberg procedure (q_fdr_bh) to control the false discovery rate (FDR). Effect sizes were additionally quantified using Cliff’s delta (δ) and interpreted as negligible, small, medium, or large according to established thresholds. Biological significance was further assessed by calculating log_2_ fold change (log_2_FC) with thresholds of ±0.2, ±0.5, and ±1.0. All data processing and statistical analyses were performed using Python v3.11 (Python Software Foundation, Wilmington, DE, USA) (pandas, numpy, scipy, statsmodels open-source libraries), the factor_analyzer library for reliability and factor loadings, and Microsoft Excel 365 (Microsoft Corp., Redmond, WA, USA).

### 2.5. Profile and Sub-Profile Formation

Raw data were collected in Excel spreadsheets (Supplementary Table S1, Final dataset 2023–2025) and included a spectrum of clinical, functional, biochemical, instrumental, and questionnaire-derived variables. Each row represented an individual patient, and each column corresponded to a specific marker linked to the year of observation. From the complete dataset, three system-specific profiles were constructed: pulmonary, cardiac, and renal.

Pulmonary profile (26 markers): Lactate, SaO_2_ before and after the 6MWT, pCO_2_, pO_2_, diastolic blood pressure before and after 6MWT, 6MWT distance, FEV_1_, lung volume, systolic blood pressure before and after 6MWT, exhaled CO_2_, FVC, respiratory rate (before and after 6MWT), heart rate (before and after 6MWT), Borg scale (before and after 6MWT), CT densitometry metrics, fibrosis density, change in respiratory rate during 6MWT. The internal consistency coefficients of the pulmonary profile in patients with SSc-ILD and COPD are presented in Table 1.

Cardiac profile (8 markers): LVEF, heart rate variability indices (HF, LF, VLF, TP), MR-proANP, hsTnT, NT-proBNP. The internal consistency coefficients of the cardiac profile in patients with SSc-ILD and COPD are presented in Table 2.

Renal profile (5 markers): eGFR, serum creatinine, albumin-to-creatinine ratio, venous sodium, renal CT densitometry.

A dedicated dictionary was created to assign each marker to one of the three system profiles. All variables were standardized to the same polarity, such that higher values indicated worse status. Variables with bidirectional deviations from reference ranges (pH, pO_2_, pCO_2_, lactate, exhaled CO_2_) were flagged separately. Standardization was performed using z-scores (subtracting the sample mean and dividing by the standard deviation), ensuring data centering (μ = 0) and unit variance (σ = 1), which allowed comparison across markers with different measurement scales.

Cronbach’s α values indicated acceptable internal consistency of the pulmonary profile in patients with SSc-ILD, whereas the corresponding values in COPD were low. This finding was supported by McDonald’s ω, which ranged from 0.271 to 0.356 across annual COPD cohorts, with an overall ω of 0.243 for COPD compared to 0.548 for SSc-ILD.

Principal component analysis (PCA) of the pulmonary profile (Supplementary Table S2) showed that PC1 explained 33% of the variance, PC2 15%, PC3 6%, and PCs 4–5 approximately 6% each. Based on these results, the pulmonary profile was initially divided into the following sub-profiles (Supplementary Table S3):

P1: FVC, FEV_1_.

P2: pO_2_, pCO_2_, pH, lactate, exhaled CO_2_, baseline SaO_2_ (6MWT).

P3: 6MWT distance, post-6MWT SaO_2_, post-6MWT Borg score, post-6MWT respiratory rate.

P4: lung volume, fibrosis density, CT densitometry.

A repeated PCA (Supplementary Table S4) refined this structure, yielding narrower sub-profiles:

R1: FVC, FEV_1_.

R2: pO_2_.

R3: pCO_2_.

R4: pH.

R5: lactate.

R6: exhaled CO_2_.

R7: baseline SaO_2_ and post-6MWT SaO_2_.

R8: 6MWT distance.

R9: post-6MWT Borg score and respiratory rate.

R10: lung volume.

R11: CT densitometry, fibrosis density.

Cronbach’s α and McDonald’s ω coefficients in COPD patients were 0.06 and 0.02, respectively, indicating poor internal consistency; similar findings were observed in SSc-ILD patients (α = 0.11, ω = 0.35). Year-by-year analysis showed that indices in COPD remained consistently low (α = 0.41–0.18–0.13; ω = 0.06–0.08–0.06), whereas in SSc-ILD values were relatively higher and more stable (α = 0.11–0.21–0.21; ω = 0.001–0.46–0.43). Although coefficients in SSc-ILD were greater than in COPD, they still indicated limited internal consistency. Overall, Cronbach’s α was 0.08, and McDonald’s ω was 0.003, both reflecting poor reliability of the cardiac profile. Therefore, additional analyses were performed, including “Alpha if item deleted” and “delta_alpha” calculations for each marker of the cardiac profile.

All HRV markers were above the overall Cronbach’s α coefficient of the cardiac profile. MR-proANP and hsTnT were slightly below the overall value, whereas LVEF, NT-proBNP, and sPAP demonstrated negative contributions. Based on these results, a corrected cardiac profile consisting of five markers (MR-proANP, hsTnT, LVEF, NT-proBNP, and sPAP) was defined. A repeated Cronbach’s α analysis of this adjusted profile yielded a coefficient of 0.40.

To further explore a unique composite indicator for the cardiac profile, principal component analysis (PCA) was performed (Supplementary Table S2). PC1 and PCs 4–5 showed the strongest contributions from NT-proBNP, sPAP, and LVEF, while PC2 and PC3 were predominantly influenced by MR-proANP and hsTnT. This allowed identification of three sub-classes of the cardiac profile:

C1: hsTnT, sPAP, LVEF

C2: NT-proBNP, MR-proANP

C3: HRV indices (LF, HF, TP)

Given the low values of VLF and IN, these markers were excluded. A repeated PCA (Supplementary Table S4) further refined the structure, resulting in four cardiac sub-profiles:

C1: hsTnT, sPAP.

C2: LVEF.

C3: MR-proANP, NT-proBNP.

C4: HRV indices (LF, HF, TP).

The renal profile markers demonstrated moderate internal consistency (Cronbach’s α = 0.64, McDonald’s ω = 0.63), while in the SSc-ILD group McDonald’s ω was lower (0.14). Principal component analysis (PCA) was performed, and the nephrology profile was divided into two sub-profiles (Supplementary Table S4). Supplementary Table S5 summarizes the final profiles and sub-profiles and their designations.

The overall results of internal consistency coefficients are presented in Table 3.

As shown in Table 3, all profiles demonstrated acceptable internal consistency. The only exception was the nephrology profile in COPD patients, where Cronbach’s α reached 0.53 (moderate consistency), while McDonald’s ω was 0.78, lower than in SSc-ILD. This discrepancy is likely explained by the heterogeneous, bidirectional behavior of markers. When evaluating each marker with the “alpha if deleted” approach, the overall index remained unchanged (Supplementary Table S6), probably due to the divergent dynamics of the markers that affected the cumulative integral index during the calculation of Cronbach’s α. All sub-profiles demonstrated sufficient internal consistency except for heart rate variability, which requires cautious interpretation.

For each sub-profile, indicators were combined into a single composite measure by calculating the mean value of all standardized variables included in the sub-profile (mean-z). Thus, each sub-profile reflected the generalized state of a functional system based on a group of closely related parameters. To confirm that the selected markers within each sub-profile indeed reflected the same construct, additional internal consistency checks were performed. Internal consistency was evaluated using Cronbach’s α and McDonald’s ω (based on PCA, ML, and MINRES approaches). Furthermore, exploratory factor analysis (EFA) was applied to examine factor structure and identify latent constructs. Factors were extracted using principal axis factoring and maximum likelihood methods. The number of factors was determined according to the Kaiser criterion (eigenvalue > 1). Data adequacy was verified using the Kaiser–Meyer–Olkin (KMO) index and Bartlett’s test of sphericity. Factor loadings ≥ 0.40 were considered significant, confirming the aggregation of markers into a common sub-profile (Supplementary Table S7).

In the final configuration, all sub-profiles were integrated into three major profiles:

Cardiac (C1–C4)

Renal (N1–N2)

Pulmonary (R1–R11)

Correlations between profiles were assessed using Spearman’s coefficient.

Principal component analysis (PCA) was applied to identify clusters of interrelated variables and to reduce data dimensionality, allowing the formation of system-specific sub-profiles with minimal redundancy. Cronbach’s α and McDonald’s ω were used to evaluate the internal consistency of each sub-profile: α for simpler unidimensional structures and ω for multidimensional constructs. This combined psychometric–biomedical approach has been increasingly applied in translational studies to verify construct validity and ensure that grouped biomarkers reflect coherent physiological processes rather than statistical artifacts.

## 3. Results

### 3.1. Dynamics of Profile Changes in Patients with SSc-ILD and COPD

A prospective follow-up was performed in two cohorts (SSc-ILD and COPD) across three visits (2023, 2024, and 2025). Tables presenting the longitudinal changes in clinical and laboratory markers for both groups over the three-year period are provided in Supplementary Table S8. The subsequent sections describe the dynamics of individual markers in each cohort and highlight between-group differences.

#### 3.1.1. Pulmonary Profile Indicators

Six-minute walk distance (6MWD). At baseline, the 6MWD was significantly lower in COPD compared with SSc-ILD (245 m [180–320] vs. 300 m [235–358]; between-group *p* = 0.02; q = 0.04). Over time, both cohorts demonstrated a decline: by 2025 the reduction reached −10 m in SSc-ILD (*p* = 0.006; q = 0.01) and −20 m in COPD (*p* = 0.002; q = 0.004). Between-group changes were not statistically significant and had a small effect size (*p* = 0.402; q = 0.676; δ = 0.089).

Pre-exercise oxygen saturation (SaO_2_). Baseline SaO_2_ was higher in SSc-ILD (98% [95.5–98]) than in COPD (95.5% [94–96]) (*p* < 0.001; q < 0.001). From 2024 to 2025, a moderate decline was observed in both cohorts (SSc-ILD: *p* = 0.001; q = 0.002; COPD: *p* < 0.001; q < 0.001). Between-group Δ2025–2024 did not reach significance after FDR correction and had a small effect size (*p* = 0.029; q = 0.187; δ = 0.216). The overall Δ2025–2023 was also non-significant (*p* = 0.52; q = 0.749; δ = 0.066).

Post-exercise oxygen saturation. In 2023, post-6MWT SaO_2_ was lower in COPD (89.5% [88–94.8]) compared to SSc-ILD (95% [93.5–97]) (*p* < 0.001; q < 0.001). Desaturation progressed in both cohorts during 2024–2025 (both *p* < 0.001; q < 0.001). Between-group Δ2025–2024 and Δ2025–2023 remained non-significant and small (*p* = 0.504; q = 0.749; δ = 0.067; and *p* = 0.313; q = 0.62; δ = 0.105, respectively).

Subjective dyspnea (Borg scale). Pre-exercise Borg scores were higher in COPD in 2024 (*p* = 0.01; q = 0.03). By 2025, both groups showed a significant increase vs. 2024 (both *p* < 0.001; q < 0.001). Between-group Δ2025–2024 was small (*p* = 0.028; q = 0.187; δ = −0.186), as was the total Δ2025–2023 (*p* = 0.035; q = 0.187; δ = −0.195). Post-exercise dyspnea was consistently higher in COPD in 2024–2025 (*p* ≤ 0.01; q ≤ 0.125), with a moderate between-group effect size for the total Δ2025–2023 (δ = −0.433).

Respiratory and heart rate, blood pressure. HR and RR increased by 2025 both at rest and post-6MWT (within-group *p* < 0.001; q < 0.001). Both systolic and diastolic BP after 6MWT also rose significantly in both cohorts (*p* < 0.001; q ≈ 0.001), with no meaningful between-group differences (|δ| < 0.20).

FEV_1_. At baseline, SSc-ILD patients had higher FEV_1_ than COPD (*p* < 0.001; q < 0.001). From 2024→2025, both groups showed a decline (*p* < 0.001; q < 0.001). The between-group Δ2025–2024 was non-significant (*p* = 0.067; q = 0.233; δ = −0.20). Over three years, the decline was greater in COPD (*p* = 0.02; q = 0.031), while in SSc-ILD it was not significant (*p* = 0.551; q = 0.599). The between-group effect remained small (*p* = 0.171; q = 0.426; δ = 0.15).

FVC. No baseline differences were found (*p* = 1.000; q = 1.000). Both cohorts declined in 2024→2025 (*p* < 0.001; q < 0.001). Between-group Δ2025–2024 was negligible (δ = −0.065). Across 2023–2025, the decline was significant in COPD (*p* < 0.001; q < 0.001), with a small between-group effect (*p* = 0.004; q = 0.087; δ = 0.31).

Lung volume (CT). Higher at baseline in COPD (*p* < 0.001; q < 0.001). In SSc-ILD, sequential reduction was seen in 2023→2024 (*p* = 0.005; q = 0.009) and 2024→2025 (*p* = 0.0001; q = 0.0002). In COPD, the 2024→2025 change was not significant (*p* = 0.41; q = 0.44). Between-group Δ2025–2024 was small (*p* = 0.053; q = 0.22; δ = −0.204).

Lung densitometry (fibrosis density). In SSc-ILD, progressive densification was observed from 2024→2025 (less negative HU; *p* = 0.002; q = 0.003), with a trend over 2023–2025 (*p* = 0.056; q = 0.083). COPD showed no significant change (2025 vs. 2024: *p* = 0.374; q = 0.44). The between-group difference at 2025 approached significance with a small-to-moderate effect (*p* = 0.04; q = 0.06; δ ≈ 0.28–0.30).

Lactate. Consistent increase in both cohorts, significant over 2024→2025 and 2023→2025 (all *p* < 0.001; q < 0.001). Between-group differences were absent (*p* ≈ 0.99; q ≈ 0.996; δ ≈ 0.001).

pCO_2_. Moderate year-to-year increase in both groups (2024 vs. 2023: *p* ≤ 0.003; q ≤ 0.005; 2025 vs. 2024: *p* < 0.001; q < 0.001). Between-group Δ2025–2023 was non-significant and small (*p* = 0.676; q = 0.853; δ = −0.045).

pO_2_. Progressive decrease at each interval in both cohorts (*p* < 0.001; q < 0.001). Between-group differences were absent (2025–2023: *p* = 0.473; q = 0.735; δ = −0.077).

pH. Slight shift toward alkalosis in both groups, statistically significant but clinically negligible (*p* < 0.001; q < 0.001). No between-group differences (2025–2024: *p* = 0.815; q ≈ 0.815–0.84; δ ≈ −0.056).

Exhaled CO_2_. Significant decline from 2023→2024 in both groups (*p* < 0.001; q < 0.001). In 2025, the trend persisted in SSc-ILD (*p* = 0.001; q = 0.003), whereas in COPD the 2025–2024 shift was not significant (*p* = 0.208; q = 0.268). Between-group Δ2025–2023 was small (*p* = 0.137; q = 0.367; δ = −0.155).

Thus, both cohorts demonstrated an overall decline in pulmonary function, with a relatively more pronounced cumulative reduction in COPD (particularly in FVC), whereas SSc-ILD was characterized by more evident structural changes on CT (volume loss and parenchymal densification). In both groups, deterioration of gas exchange was observed (decreased pO_2_, increased pCO_2_ and lactate) without significant between-group differences, reflecting a common “systemic” signal of progression. These changes coincided with reduced exercise tolerance as assessed by the six-minute walk test.

#### 3.1.2. Cardiac Profile Parameters

Left ventricular ejection fraction (LVEF). A moderate year-to-year decline was observed in both cohorts: 2024 vs. 2023—SSc-ILD: *p* = 0.045; q = 0.066; COPD: *p* = 0.318; q = 0.350; 2025 vs. 2024—both *p* = 0.0000; q = 0.0000. No between-group differences were detected at individual timepoints (e.g., 2025: *p* = 0.163; q = 0.276).

Estimated systolic pulmonary artery pressure (sPAP). Baseline values were comparable. By 2025, a significant increase was recorded in both groups (both *p* = 0.0000; q = 0.0000), with a between-group difference favoring higher values in COPD (*p* = 0.007; q = 0.0328).

Heart rate variability (HRV). Total power and the HF component decreased by 2025 in both cohorts (within-group *p* = 0.0000; q = 0.0000). In 2025, a between-group difference in HF persisted (*p* = 0.0003; q = 0.0084), indicating differences in autonomic regulation.

NT-proBNP. Levels significantly increased by 2025 in both groups (both *p* < 0.001; q < 0.001). The between-group difference at 2025 was not significant (*p* = 0.0788; q = 0.1999). The overall Δ2025–2023 showed a significant rise in both cohorts (both *p* < 0.001; q < 0.001), with no between-group differences (q ≈ 0.613).

MR-proANP. Levels were consistently higher in SSc-ILD in 2023, 2024, and 2025 (*p* = 0.0050/0.0035/0.0026; q = 0.0276/0.0276/0.0276). Within groups, levels significantly increased by 2025 (both *p* < 0.001; q < 0.001). No between-group differences in Δ were detected (q ≈ 0.673).

hs-TnT. In 2024, a significant increase was observed in COPD (*p* = 0.0108; q = 0.0183) but not in SSc-ILD (*p* = 0.6184; q = 0.6802). By 2025, the overall Δ2025–2023 was significant in both groups (both *p* ≤ 0.0001), with no between-group differences (q ≈ 0.669).

Cardiac burden increased in both cohorts, reflected by elevated natriuretic peptides, reduced LVEF, and decreased HRV reserves. By 2025, sPAP was higher in COPD, whereas MR-proANP remained consistently higher in SSc-ILD, highlighting distinct patterns of vascular–neurohumoral dysregulation.

#### 3.1.3. Renal Profile Indicators

eGFR. A sequential decline was observed in both groups: by 2024 (SSc-ILD: *p* = 0.0008; q = 0.0014; COPD: *p* = 0.0020; q = 0.0029) and further by 2025 (both *p* < 0.001; q < 0.001). No between-group differences were found for Δ changes.

Serum creatinine. A significant increase was recorded by 2024 in both cohorts (SSc-ILD: *p* = 0.0001; q = 0.0002; COPD: *p* = 0.0035; q = 0.0047) and continued to rise in 2025 (both *p* < 0.001; q < 0.001). Serum creatinine was consistently higher in COPD (2023: *p* < 0.001; q < 0.001; 2025: *p* = 0.0001; q = 0.0005).

Albuminuria and ACR. Urinary albumin and the albumin/creatinine ratio (ACR) significantly increased by 2025 in both cohorts (both *p* < 0.001; q < 0.001). No between-group differences were detected either by year or by Δ.

Urinary creatinine. A significant within-group increase was observed by 2025 (both *p* < 0.001; q < 0.001). Levels were higher in COPD (2023: *p* = 0.0001; q = 0.0004; 2025: *p* = 0.0117; q = 0.0352). Δ2025–2023 showed growth in both groups, with no between-group differences (q ≈ 0.835).

Renal parenchymal CT densitometry (HU). Right kidney: no dynamics were detected in SSc-ILD; a slight reduction was found in COPD (Δ2025–2024 −1.00 [−2.00; 0.00], *p* = 0.0017; q = 0.0029; cumulative Δ2025–2023 −1.00). By 2025, the between-group difference disappeared (*p* = 0.8470; q = 0.9251). Left kidney: a decline was observed in both groups from 2025–2024 (both *p* ≤ 0.0008; q < 0.001). Over Δ2025–2023, the reduction was −2.00 in both cohorts (SSc-ILD: *p* < 0.001; q = 0.0227; COPD: *p* < 0.001; q < 0.001), with no between-group differences (q ≈ 1.000).

Sodium (Na). From 2024 to 2025, a slight increase was noted in both cohorts (SSc-ILD: +1.00 [−1.00; 2.00], *p* = 0.0151; q = 0.2394—non-significant after FDR; COPD: +1.00 [−1.00; 2.00], *p* = 0.0018; q = 0.0029). No significant changes were found for Δ2025–2023 (both q ≥ 0.367). Clinical significance was minimal.

The renal axis in both diseases demonstrated early degradation (declining eGFR, increasing creatinine, and rising ACR) without substantial between-group differences in progression rates; however, creatinine levels (both serum and urinary) were higher in COPD at key timepoints. Changes in HU and Na were of secondary clinical relevance.

### 3.2. Integrated Profile Metrics

#### 3.2.1. Dynamics of Changes Across Profiles and Subprofiles

Table 4 presents the summary analysis of profiles in patients with COPD and SSc-ILD over the three-year follow-up period.

According to Table 4, pronounced between-group differences were observed mainly at baseline and in different domains, with varying directions. The cardiac profile was worse in SSc-ILD in 2023 (Cliff’s δ ≈ 0.27; *p* ≈ 0.0107; q ≈ 0.0479; biological significance—small to moderate). However, in 2024 the effect weakened and did not reach significance (δ ≈ 0.18; q ≈ 0.25), and by 2025 the differences had disappeared (δ ≈ 0.07; q ≈ 0.72). Considering the integrative profile indicator, a subprofile analysis was subsequently performed (Table 5). In subprofile C1, a negative trend was observed in COPD patients, largely due to an increase in sPAP, particularly by the third year of follow-up. In subprofile C2, a negative trajectory was noted in both groups, but SSc-ILD patients had initially lower LVEF values and showed greater rates of decline over time. In subprofile C3, SSc-ILD patients demonstrated an increase in heart failure markers (MR-proANP, ntproBNP), while values remained relatively stable in COPD. In subprofile C4, HRV parameters (LF, HF, TP) were worse in COPD, suggesting a disruption in heart rate regulation mechanisms.

The renal profile demonstrated the largest baseline gap: in 2023, values were substantially worse in SSc-ILD (δ ≈ 0.69; *p* ≈ 0; q ≈ 0; large effect). Subsequently, between-group differences were attenuated (2024: δ ≈ 0.04; q ≈ 0.72), and by 2025 a shift toward less favorable values was observed in COPD (δ ≈ −0.10; q ≈ 0.72), although not statistically significant. Subprofile analysis indicated that COPD patients showed more pronounced deviations in renal functional status (eGFR, serum creatinine, urinary creatinine, albumin/creatinine ratio; N1), while SSc-ILD patients had more marked CT-densitometric indicators of renal fibrosis (N2).

For the pulmonary profile, no significant between-group differences were observed at annual timepoints (|δ| ≤ 0.07; q ≥ 0.64). The direction of the small effects varied, and by 2025 values were slightly worse in COPD (δ ≈ −0.05; q ≈ 0.72). Subprofile results were as follows: in R1, more pronounced changes in FVC and FEV_1_ across all years were recorded in COPD, with relatively stable values in SSc-ILD. In R2 and R3, COPD patients consistently demonstrated a more unfavorable picture of hypoxemia and hypercapnia. In R4, the majority of COPD patients exhibited blood alkalosis (pH shift). In R5, serum lactate elevations in 2023 were more pronounced in COPD, but no major differences were observed in later years within or between groups. In R6 (exhaled CO_2_), more marked changes were noted in SSc-ILD. Subprofile R7 indicated more severe desaturation both before and after the 6MWT in COPD patients. Consistently, in R8 and R9, COPD patients showed shorter walking distances during the 6MWT and higher dyspnea scores. In R10 and R11, SSc-ILD patients demonstrated more pronounced CT-densitometric features of lung fibrosis (reduced lung volumes, parenchymal densification).

#### 3.2.2. Interrelationships Between Profiles

Correlation analysis is presented in Table 6. Weak associations were observed between all profiles. This is likely explained by the relative autonomy of impairments within the cardio–pulmo–renal continuum and supports the use of multichannel monitoring rather than reliance on a single domain.

Detailed pairwise correlations between subprofiles are presented in Table 7.

Strong positive correlations were identified between subprofiles: R1 (FVC; FEV_1_) and R7 (SaO_2_ pre-6MWT; SaO_2_ post-6MWT); as well as between R7 (SaO_2_ pre-6MWT; SaO_2_ post-6MWT) and R9 (Borg scale post-6MWT; RR post-6MWT).

Moderate positive correlations were observed between the following subprofiles: C1 (hs-TnT; sPAP) and R1 (FVC; FEV_1_); C1 (hs-TnT; sPAP) and R7 (SaO_2_ pre-6MWT; SaO_2_ post-6MWT); C3 (MR-proANP, NT-proBNP) and R10 (lung volume); N2 (renal densitometry) and R10 (lung volume); R1 (FVC; FEV_1_) and R8 (6MWT distance); R1 (FVC; FEV_1_) and R9 (Borg scale post-6MWT; RR post-6MWT); R10 (lung volume) and R11 (CT densitometry; fibrosis density); R7 (SaO_2_ pre-6MWT; SaO_2_ post-6MWT) and R8 (6MWT distance); R8 (6MWT distance) and R9 (Borg scale post-6MWT; RR post-6MWT).

Moderate negative correlations were found between subprofiles: N1 (eGFR; serum creatinine; urinary creatinine; albumin/creatinine ratio) and R6 (exhaled CO_2_); C3 (MR-proANP, NT-proBNP) and C4 (HRV: LF, HF, TP); N2 (renal densitometry) and R1 (FVC; FEV_1_); R1 (FVC; FEV_1_) and R10 (lung volume); R10 (lung volume) and R7 (SaO_2_ pre-6MWT; SaO_2_ post-6MWT); R10 (lung volume) and R9 (Borg scale post-6MWT; RR post-6MWT); R2 (pO_2_) and R3 (pCO_2_); R3 (pCO_2_) and R4 (pH).

## 4. Discussion

This 3-year longitudinal analysis revealed disease-specific trajectories within the pulmo–cardio–renal continuum. Patients with SSc-ILD exhibited predominantly fibrotic and cardiac remodeling patterns, while those with COPD showed progressive hemodynamic overload and renal impairment [18,19]. These multidimensional trajectories reflect different systemic pathways leading from primary pulmonary injury toward secondary cardiac and renal dysfunction.

Rather than isolated organ manifestations, these findings emphasize the systemic nature of chronic lung diseases and the integrative interplay among respiratory, cardiovascular, and renal domains. The observed inter-profile associations suggest that structural and functional pulmonary changes may trigger downstream alterations through shared mechanisms of hypoxemia, endothelial dysfunction, neurohormonal activation, and microvascular remodeling.

These findings were further interpreted in the context of existing evidence, as discussed below.

Pulmo–cardiac interactions. Moderate positive associations between hsTnT, sPAP, spirometric indices (FVC, FEV_1_), and oxygen saturation both before and after exercise may reflect manifestations of pulmonary dysfunction linked to the progression of cardiac impairment. Kvisvik et al. reported that elevated hs-cTnT levels were associated with the severity of pulmonary hypertension and cardiac dysfunction in patients with stable COPD, as well as being an independent predictor of all-cause mortality [20,21,22]. Filusch and colleagues demonstrated that elevated hsTnT correlated with adverse outcomes, including death, and with other markers of disease severity such as reduced exercise tolerance and right ventricular dysfunction in patients with pulmonary arterial hypertension [4,23,24].

Positive correlations between natriuretic peptides (MR-proANP, NT-proBNP) and CT-derived lung volumes indicate that increased lung volumes (e.g., due to emphysematous changes) are closely linked to neurohormonal activation and cardiac overload. A meta-analysis assessing NT-proBNP levels in patients with COPD showed that NT-proBNP levels were significantly higher in patients with COPD combined with pulmonary hypertension and heart failure (HF) compared with those with COPD alone, whereas in patients with HF combined with COPD, NT-proBNP levels did not differ significantly from those in patients with isolated HF [4,25]. The study also demonstrated significant differences in NT-proBNP levels between survivors and non-survivors during hospitalization for COPD exacerbation. The authors concluded that chronic hypoxemia and structural remodeling of the pulmonary circulation in COPD disrupt cardio-pulmonary interactions and trigger remodeling of the right atrium and ventricle, as reflected by increased NT-proBNP. Moreover, airway inflammation plays a crucial role in the pathogenesis of COPD, with immune cell activation and the release of pro-inflammatory mediators extending beyond the lungs to neighboring organs, thereby further contributing to elevated NT-proBNP [26].

The interpretation of HRV alterations requires caution, as these indices reflect complex autonomic interactions rather than linear changes in sympathetic or parasympathetic tone. Reduced LF power in SSc-ILD may indicate baroreflex–cardiovagal insufficiency secondary to pulmonary hypertension, hypoxemia, or lung stiffness, whereas increased HF power in COPD may represent compensatory parasympathetic activation during disease exacerbation. Similar mechanisms have been reported in studies linking baroreflex dysfunction with pulmonary hyperinflation and autonomic imbalance in chronic lung disease [27,28].

These observations suggest that HRV markers may capture cohort-specific physiological variability. However, further longitudinal validation is needed to establish their predictive and translational value in chronic pulmonary disorders.

Pulmo-renal interactions. Moderate positive correlations between CT-derived kidney densitometry values and CT-based lung volumes reflect the interrelationship of structural changes in the respiratory and renal systems. This may be attributed to shared pathophysiological mechanisms, including fibrosis, systemic inflammation, and microcirculatory disturbances. Chronic inflammation in COPD triggers the release of pulmonary and systemic inflammatory mediators, including tumor necrosis factor-α (TNF-α), interleukin-6 (IL-6), and C-reactive protein (CRP) [29,30]. Elevated CRP levels have been shown to correlate positively with the onset and progression of chronic kidney disease (CKD), suggesting that chronic inflammation may impair renal function by affecting tubular epithelial cells and glomeruli [31]. TNF-α induces apoptosis of tubular epithelial cells and promotes renal interstitial fibrosis through activation of the nuclear factor κB (NF-κB) signaling pathway. Thus, inflammation-mediated apoptosis and fibrotic remodeling may play parallel roles in systemic inter-organ sclerosis, leading to dysfunction of one or both organs.

Negative associations between renal function markers (eGFR, creatinine) and exhaled CO_2_, as well as between renal densitometry and spirometric indices, suggest that impaired kidney function is associated with more pronounced disturbances in gas exchange and ventilation. In early COPD, renal perfusion is usually preserved; however, as the disease progresses—particularly with the development of CO_2_ retention—renal blood flow declines. PaCO_2_ has been shown to correlate inversely with effective renal plasma flow (ERPF) and with the ability to excrete sodium and water. Hypercapnia may induce renal vasoconstriction both directly and indirectly by stimulating sympathetic tone, as reflected by increased circulating norepinephrine levels [32]. With progression of pulmonary insufficiency, systemic hypoxia and hypercapnia intensify, further impairing the kidneys through mechanisms of chronic ischemia and activation of the renin–angiotensin–aldosterone system (RAAS). It is well established that RAAS plays a key role in stimulating pro-inflammatory mediators such as IL-6 and TNF-α. This cascade promotes collagen synthesis, which contributes to the pathogenesis of both pulmonary and renal fibrosis.

The identified inter-profile correlations underscore the systemic nature of the pulmo–cardio–renal interactions. Pulmonary dysfunction exerts a cascading impact on the heart and kidneys, reflected in concordant changes in biomarkers, functional indices, and structural parameters. These findings support the concept of the “lung as the initiating organ” in the development of multiorgan dysfunction.

They also demonstrate that when primary pulmonary impairment is present within its underlying nosology, a pathological cascade is triggered that involves other systems—in this case, the heart and kidneys—allowing this process to be framed within the pulmo–cardio–renal continuum.

This study has several limitations that should be taken into account when interpreting the findings and designing future research. First, the limited sample size and single-center design reduce the generalizability of the results to broader populations. External validation has not yet been performed; however, several levels of internal validation were implemented, including internal consistency within profiles, cross-validation between subprofiles, and correlation analyses between biomarkers, which support the internal robustness of the findings. Second, imbalances between groups with respect to age, sex, smoking status, and concomitant therapy, as well as attrition over the three-year follow-up, may have introduced bias. In addition, potential confounders such as age, comorbidities, and medication use were not statistically adjusted for, which may have influenced the magnitude of associations observed. Third, the inclusion criterion of baseline eGFR ≥ 60 mL/min/1.73 m^2^ restricted the potential study population. However, this threshold was intentionally applied to minimize bias and to obtain a more objective evaluation of early renal changes without the confounding influence of preexisting advanced chronic kidney disease. Fourth, the influence of treatment (e.g., inhaled corticosteroids, antifibrotic agents, ACE inhibitors/ARBs, diuretics) and comorbidities (e.g., diabetes mellitus, arterial hypertension, ischemic heart disease) may have modified the studied markers and their interrelations. Fifth, the observational design and correlational analysis do not allow causal inference; the use of composite scores and PCA improves metric robustness but limits direct clinical interpretability.

Finally, the analysis was deliberately restricted to the pulmo–cardio–renal axis, which may obscure the contribution of other systemic interactions (e.g., cardio-metabolic, neurovegetative, hepato-renal, musculoskeletal). Additionally, HRV results should be interpreted with caution, given their relatively low consistency across subprofiles.

Future studies should consider expanding the analytical framework by incorporating cardio-metabolic markers, systemic inflammation profiles, and musculoskeletal parameters. Furthermore, evaluating the dynamics of profiles and subprofiles in the context of therapeutic interventions in COPD and SSc-ILD populations may help to disentangle treatment effects from disease-related progression.

## 5. Conclusions

In this 3-year longitudinal observation of patients with COPD and SSc-ILD, a possible initiation of inter-organ alterations was identified against the background of pulmonary involvement, leading to the formation of a pulmo–cardio–renal continuum with distinct disease-specific trajectories. In COPD, hyperinflation and impaired gas exchange predominated, followed by rising systolic pulmonary artery pressure and disruption of heart rate variability by year 3. COPD patients also demonstrated the most pronounced decline in eGFR and increase in albuminuria. In SSc-ILD, cardiac dysfunction and structural CT markers of fibrosis—both pulmonary and renal—were more prominent.

Inter-profile correlations confirmed systemic interconnectedness. Unidirectional associations indicated common mechanisms (chronic hypoxemia/hypercapnia, neurohormonal activation, microvascular impairment, and fibrosis) through which pulmonary dysfunction drives cardiac and renal impairment. Clinically, these findings support the need for multi-organ monitoring (lung volumes/gas exchange, NT-proBNP/hsTnT, systolic pulmonary artery pressure, eGFR/ACR, and renal/pulmonary CT densitometry) and the use of composite/sub-profile indices as sensitive integrative markers of disease dynamics. The results reinforce the concept of the lung as an initiating organ and provide a rationale for personalized monitoring and therapy, emphasizing intensified cardio-renal surveillance in SSc-ILD and nephroprotective strategies in COPD based on composite profile assessment.

## Figures and Tables

**Table 1:** Internal consistency coefficients of the pulmonary profile in patients with SSc-ILD and COPD.

Profile	Markers	Group	Cronbach’s α	McDonald’s ω
Pulmonary profile	lactate, SaO_2_ before and after the 6MWT, pCO_2_, pO_2_, diastolic blood pressure before and after 6MWT, 6MWT distance, FEV_1_, lung volume, systolic blood pressure before and after 6MWT, exhaled CO_2_, FVC, respiratory rate (before and after 6MWT), heart rate (before and after 6MWT), Borg scale (before and after 6MWT), CT densitometry metrics, fibrosis density, change in respiratory rate during 6MWT	COPD 2023	0.338	0.271
		COPD 2024	0.22	0.261
		COPD 2025	0.34	
		SSc-ILD 2023	0.452	0.356
		SSc-ILD 2024	0.529	
		SSc-ILD 2025	0.565	
		group_COPD	0.691	0.243
		group_SSc-ILD	0.819	0.548
		Overall score	0.76	0.501

**Table 2:** Internal consistency coefficients of the cardiac profile in patients with SSc-ILD and COPD.

Profile	Markers	Group	Cronbach’s α	McDonald’s ω
Cardiac profile	LVEF, systolic pulmonary artery pressure (sPAP), HRV indices (IN, HF, LF, VLF, TP), NT-proBNP, MR-proANP, hsTnT	COPD 2023	0.41	0.06
		COPD 2024	0.18	0.08
		COPD 2025	0.13	0.06
		SSc-ILD 2023	0.11	0.001
		SSc-ILD 2024	0.21	0.46
		SSc-ILD 2025	0.21	0.43
		group_COPD	0.06	0.02
		group_SSc-ILD	0.11	0.35
		Overall score	0.08	0.003

**Table 3:** Internal consistency coefficients (Cronbach’s α and McDonald’s ω) of cardiac, renal, and pulmonary profiles and sub-profiles in patients with SSc-ILD, COPD, and in the overall cohort.

Profile	Group	Cronbach’s α	McDonald’s ω	Subprofiles	Markers	Group	Cronbach’s α	McDonald’s ω
Cardiac profile	SSC-ILD	0.78	0.82	C1	hsTnT; sPAP	SSC-ILD	0.74	0.81
						COPD	0.72	0.78
						All	0.72	0.79
				C2	LVEF	SSC-ILD	0.76	0.87
	COPD	0.72	0.78			COPD	0.75	0.86
						All	0.76	0.86
				C3	MR-proANP. ntproBNP	SSC-ILD	0.86	0.93
						COPD	0.98	0.99
	All	0.66	0.74			All	0.96	0.97
				C4	HRV indices (LF. HF. TP)	SSC-ILD	0.39	0.18
						COPD	0.25	0.04
						All	0.32	0.07
Pulmonary profile	SSC-ILD	0.92	0.93	R1	FVC; FEV_1_	SSC-ILD	0.97	0.98
						COPD	0.86	0.91
						All	0.9	0.93
				R2	pO_2_	SSC-ILD	0.94	0.96
						COPD	0.91	0.95
						All	0.93	0.95
				R3	pCO_2_	SSC-ILD	0.9	0.94
	COPD	0.92	0.93			COPD	0.94	0.96
						All	0.93	0.96
				R4	pH	SSC-ILD	0.87	0.92
						COPD	0.89	0.93
						All	0.88	0.93
				R5	Lactate	SSC-ILD	0.95	0.97
						COPD	0.94	0.96
						All	0.95	0.97
				R6	Exhaled CO_2_	SSC-ILD	0.97	0.98
						COPD	0.98	0.99
						All	0.98	0.99
				R7	SaO_2_ before 6MWT; SaO_2_ after 6MWT	SSC-ILD	0.98	0.98
						COPD	0.95	0.96
	All	0.91	0.92			All	0.97	0.97
				R8	6MWT distance	SSC-ILD	0.97	0.98
						COPD	0.98	0.99
						All	0.98	0.98
				R9	Borg scale. respiratory rate after 6MWT	SSC-ILD	0.92	0.94
						COPD	0.88	0.91
						All	0.91	0.94
				R10	Lung volume	SSC-ILD	0.96	0.97
						COPD	0.98	0.99
						All	0.99	0.99
				R11	CT densitometry; fibrosis density	SSC-ILD	0.89	0.92
						COPD	0.83	0.88
						All	0.89	0.92
Renal profile	SSC-ILD	0.80	0.83	N1	eGFR; Serum creatinine; Urinary creatinine; ACR	SSC-ILD	0.82	0.85
						COPD	0.82	0.86
	COPD	0.53	0.78			All	0.82	0.85
				N2	Right kidney CT density; Left kidney CT densityи	SSC-ILD	0.87	0.91
	All	0.81	0.81			COPD	0.85	0.96
						All	0.86	0.9

**Table 4:** Summary analysis of profiles in patients with COPD and SSc-ILD.

Year	Profile	ILD-SSD Median [Q1–Q3] *n* = 59	COPD Median [Q1–Q3] *n* = 62	Cliff’s Delta (ILD vs. COPD)	Effect Size (Rank-Biserial r)	MWU *p*	Median Diff (ILD-SSD-COPD)	Bio Diff Bucket	q (FDR-BH)
2023	Cardiac profile	0.077 [−0.167–0.342]	−0.093 [−0.265–0.155]	0.27	0.27	0.01 *	0.169		0.048 *
2024		0.035 [−0.129–0.157]	−0.059 [−0.309–0.216]	0.184	0.184	0.082	0.094		0.246
2025		−0.007 [−0.176–0.18]	−0.087 [−0.232–0.199]	0.071	0.071	0.505	0.080		0.722
2023	Renal profile	0.217 [0.013–0.369]	−0.331 [−0.458–−0.088]	0.685	0.685	0.0008	0.548	medium	0.0007
2024		0.048 [−0.347–0.358]	0.051 [−0.337–0.302]	0.038	0.038	0.722	−0.003		0.722
2025		0.036 [−0.405–0.331]	0.096 [−0.307–0.4]	−0.101	−0.101	0.339	−0.060		0.722
2023	Pulmonary profile	−0.055 [−0.219–0.239]	−0.041 [−0.244–0.171]	0.067	0.067	0.529	−0.014		0.722
2024		−0.06 [−0.323–0.214]	−0.009 [−0.199–0.2]	−0.045	−0.045	0.673	−0.051		0.722
2025		−0.103 [−0.335–0.194]	−0.003 [−0.23–0.218]	−0.049	−0.049	0.643	−0.099		0.722

“ILD-SSD median [Q1–Q3]” and “COPD median [Q1–Q3]” represent descriptive statistics of the composites. “Cliff’s delta” and “Effect size” indicate the direction and magnitude of between-group differences (ILD-SSD relative to COPD). “MWU *p*” refers to the *p*-value from the Mann–Whitney U test; where applicable, “q” denotes the value after FDR correction. “*” indicates statistical significance (*p* < 0.05 for MWU test; *q* < 0.05 after FDR correction).

**Table 5:** Summary analysis of subprofiles in patients with COPD and SSc-ILD.

Subprofile	Year	SSc-ILD Median [Q1–Q3] *n* = 59	COPD Median [Q1–Q3] *n* = 62	Cliff’s Delta (SSc-ILD vs. COPD)	Effect Size (Rank-Biserial r)	MWU *p*	Median Diff (SSc-SSc-ILD-COPD)	Bio Diff Bucket	q (FDR-BH)
C1	2023	−0.173 [−0.475–0.32]	−0.148 [−0.41–0.493]	−0.055	−0.055	0.602	−0.024		0.672
	2024	−0.227 [−0.526–0.247]	−0.123 [−0.529–0.358]	−0.071	−0.071	0.505	−0.104		0.605
	2025	−0.326 [−0.616–0.189]	−0.1 [−0.407–0.455]	−0.214	−0.214	0.042	−0.226	small	0.090
C2	2023	0.356 [−0.336–0.702]	−0.163 [−0.855–0.529]	0.276	0.276	0.009	0.519	medium	0.024
	2024	0.254 [−0.405–0.818]	−0.123 [−0.829–0.63]	0.200	0.200	0.058	0.376	small	0.109
	2025	0.519 [−0.866–0.98]	0.057 [−0.808–0.519]	0.147	0.147	0.163	0.461	small	0.252
C3	2023	−0.217 [−0.471–0.497]	−0.406 [−0.513–−0.131]	0.237	0.237	0.025	0.188		0.056
	2024	−0.122 [−0.357–0.146]	−0.286 [−0.457–−0.028]	0.253	0.253	0.016	0.164		0.042
	2025	−0.058 [−0.281–0.167]	−0.278 [−0.479–−0.04]	0.283	0.283	0.007	0.220	small	0.023
C4	2023	−0.037 [−0.287–0.403]	0.02 [−0.35–0.511]	−0.051	−0.051	0.632	−0.057		0.686
	2024	0.137 [−0.335–0.412]	0.1 [−0.366–0.568]	−0.019	−0.019	0.860	0.037		0.860
	2025	−0.047 [−0.34–0.401]	0.066 [−0.333–0.571]	−0.061	−0.061	0.567	−0.113		0.657
N1	2023	−0.136 [−0.442–0.187]	−0.073 [−0.327–0.414]	−0.183	−0.183	0.084	−0.063		0.147
	2024	−0.154 [−0.531–0.338]	−0.018 [−0.249–0.448]	−0.166	−0.166	0.116	−0.136		0.190
	2025	−0.174 [−0.571–0.382]	−0.037 [−0.251–0.472]	−0.198	−0.198	0.061	−0.137		0.111
N2	2023	0.513 [0.513–0.513]	−0.589 [−0.589–−0.589]	0.898	0.898	0.000	1.102	large	0.000
	2024	0.141 [−0.317–0.859]	−0.17 [−0.578–0.244]	0.220	0.220	0.056	0.311	small	0.109
	2025	0.036 [−0.425–0.761]	−0.07 [−0.48–0.351]	0.060	0.060	0.606	0.106		0.672
R1	2023	−0.24 [−0.76–0.395]	0.45 [−0.549–0.805]	−0.280	−0.280	0.009	−0.690	medium	0.024
	2024	−0.308 [−0.742–0.3]	0.529 [−0.255–1.07]	−0.407	−0.407	0.000	−0.836	medium	0.001
	2025	−0.38 [−0.75–0.33]	0.494 [−0.342–1.102]	−0.390	−0.390	0.000	−0.874	medium	0.001
R10	2023	0.836 [0.527–1.125]	−0.748 [−1.174–−0.364]	0.921	0.921	0.000	1.584	large	0.000
	2024	0.806 [0.542–1.122]	−0.895 [−1.135–−0.326]	0.922	0.922	0.000	1.701	large	0.000
	2025	0.806 [0.593–1.14]	−0.849 [−1.11–−0.285]	0.912	0.912	0.000	1.655	large	0.000
R11	2023	0.198 [−0.155–0.823]	−0.425 [−0.764–0.094]	0.548	0.548	0.000	0.624	medium	0.000
	2024	0.283 [−0.172–0.792]	−0.449 [−0.674–−0.019]	0.571	0.571	0.000	0.733	medium	0.000
	2025	0.295 [−0.144–0.997]	−0.408 [−0.711–−0.026]	0.563	0.563	0.000	0.703	medium	0.000
R2	2023	−0.199 [−0.606–0.177]	−0.078 [−0.575–0.42]	−0.070	−0.070	0.510	−0.121		0.605
	2024	−0.379 [−0.634–0.256]	−0.051 [−0.699–0.515]	−0.081	−0.081	0.450	−0.328	small	0.560
	2025	−0.355 [−0.761–0.247]	−0.018 [−0.691–0.579]	−0.082	−0.082	0.444	−0.338	small	0.560
R3	2023	−0.277 [−0.642–0.54]	−0.015 [−0.865–0.923]	−0.042	−0.042	0.696	−0.263	small	0.740
	2024	−0.28 [−0.687–0.361]	−0.002 [−0.878–0.805]	−0.096	−0.096	0.369	−0.277	small	0.483
	2025	−0.283 [−0.588–0.401]	−0.039 [−0.686–0.648]	−0.097	−0.097	0.365	−0.244	small	0.483
R4	2023	−0.001 [−0.5–0.902]	0.13 [−0.934–0.671]	0.099	0.099	0.351	−0.131		0.483
	2024	0.132 [−0.419–0.649]	0.222 [−0.986–0.699]	0.031	0.031	0.776	−0.090		0.808
	2025	0 [−0.383–0.654]	0.169 [−0.856–0.722]	0.028	0.028	0.792	−0.169		0.808
R5	2023	−0.358 [−0.751–0.275]	−0.112 [−0.522–0.662]	−0.173	−0.173	0.104	−0.245	small	0.177
	2024	−0.398 [−0.775–0.221]	−0.275 [−0.615–0.66]	−0.153	−0.153	0.151	−0.123		0.241
	2025	−0.432 [−0.75–0.235]	−0.295 [−0.594–0.653]	−0.119	−0.119	0.262	−0.137		0.371
R6	2023	0.125 [−0.287–1.023]	−0.1 [−1.073–0.723]	0.209	0.209	0.049	0.225	small	0.100
	2024	0.102 [−0.293–1.07]	0.03 [−1.046–0.639]	0.143	0.143	0.176	0.072		0.265
	2025	0.082 [−0.297–1.106]	0.006 [−1.055–0.613]	0.128	0.128	0.228	0.076		0.333
R7	2023	−0.757 [−0.943–0.013]	0.14 [−0.151–0.851]	−0.460	−0.460	0.000	−0.897	medium	0.000
	2024	−0.772 [−0.983–−0.196]	0.211 [−0.4–0.801]	−0.519	−0.519	0.000	−0.983	medium	0.000
	2025	−0.675 [−0.85–−0.047]	0.259 [−0.383–0.989]	−0.486	−0.486	0.000	−0.934	medium	0.000
R8	2023	−0.21 [−0.738–0.386]	0.294 [−0.394–0.891]	−0.248	−0.248	0.019	−0.505	medium	0.046
	2024	−0.392 [−0.976–0.427]	0.252 [−0.567–1.012]	−0.277	−0.277	0.009	−0.643	medium	0.024
	2025	−0.26 [−0.91–0.434]	0.258 [−0.501–0.989]	−0.298	−0.298	0.005	−0.526	medium	0.016
R9	2023	−0.338 [−0.844–0.52]	0.216 [−0.338–0.566]	−0.236	−0.236	0.025	−0.554	medium	0.056
	2024	−0.472 [−1.147–0.24]	0.153 [−0.123–0.657]	−0.450	−0.450	0.000	−0.624	medium	0.000
	2025	−0.469 [−1.09–0.278]	0.2 [−0.217–0.704]	−0.423	−0.423	0.000	−0.669	medium	0.000

**Table 6:** Correlation analysis between profiles.

Profile	Cardiac Profile	Renal Profile	Pulmonary Profile
Cardiac profile	1	−0.10038	0.10462
Renal profile	−0.10038	1	−0.11329
Pulmonary profile	0.10462	−0.11329	1

**Table 7:** Correlation analysis between subprofiles.

Subprofiles	C1	C2	C3	C4	N1	N2	R1	R10	R11	R2	R3	R4	R5	R6	R7	R8	R9
C1	1.00	−0.19	0.12	−0.18	−0.14	−0.09	0.30 *	−0.13	0.00	0.16	0.00	−0.03	−0.01	0.10	0.30 *	0.18	0.25
C2	−0.19	1.00	0.04	0.04	−0.11	0.04	−0.04	0.16	0.06	−0.06	0.03	0.08	0.01	0.03	−0.14	−0.02	−0.08
C3	0.12	0.04	1.00	−0.33 *	−0.06	0.12	0.03	0.32 *	0.18	−0.14	0.28	−0.15	0.15	−0.10	0.03	0.17	−0.07
C4	−0.18	0.04	−0.33 *	1.00	0.04	0.13	−0.07	−0.16	−0.14	−0.13	−0.06	−0.03	0.03	0.14	−0.09	−0.12	−0.02
N1	−0.14	−0.11	−0.06	0.04	1.00	−0.02	−0.13	−0.11	0.10	−0.14	0.10	−0.10	0.10	−0.31 *	0.11	0.00	0.13
N2	−0.09	0.04	0.12	0.13	−0.02	1.00	−0.34 *	0.37 *	0.12	−0.02	0.05	−0.03	0.03	0.10	−0.24	−0.08	−0.07
R1	0.30 *	−0.04	0.03	−0.07	−0.13	−0.34 *	1.00	−0.32 *	−0.14	−0.08	0.11	0.12	0.19	−0.03	0.68 *	0.41 *	0.51 *
R10	−0.13	0.16	0.32 *	−0.16	−0.11	0.37 *	−0.32 *	1.00	0.56 *	−0.10	−0.02	0.08	−0.02	0.05	−0.41 *	−0.26	−0.33 *
R11	0.00	0.06	0.18	−0.14	0.10	0.12	−0.14	0.56 *	1.00	−0.05	−0.03	0.17	−0.03	0.09	−0.11	−0.06	0.03
R2	0.16	−0.06	−0.14	−0.13	−0.14	−0.02	−0.08	−0.10	−0.05	1.00	−0.31 *	0.21	−0.04	0.26	0.03	−0.05	0.02
R3	0.00	0.03	0.28	−0.06	0.10	0.05	0.11	−0.02	−0.03	−0.31 *	1.00	−0.56 *	0.28	−0.08	0.18	0.11	0.12
R4	−0.03	0.08	−0.15	−0.03	−0.10	−0.03	0.12	0.08	0.17	0.21	−0.56 *	1.00	−0.21	0.06	0.04	0.14	0.12
R5	−0.01	0.01	0.15	0.03	0.10	0.03	0.19	−0.02	−0.03	−0.04	0.28	−0.21	1.00	−0.02	0.22	0.25	0.20
R6	0.10	0.03	−0.10	0.14	−0.31 *	0.10	−0.03	0.05	0.09	0.26	−0.08	0.06	−0.02	1.00	−0.09	0.04	−0.01
R7	0.30 *	−0.14	0.03	−0.09	0.11	−0.24	0.68 *	−0.41 *	−0.11	0.03	0.18	0.04	0.22	−0.09	1.00	0.54 *	0.73 *
R8	0.18	−0.02	0.17	−0.12	0.00	−0.08	0.41 *	−0.26 *	−0.06	−0.05	0.11	0.14	0.25	0.04	0.54 *	1.00	0.51 *
R9	0.25	−0.08	−0.07	−0.02	0.13	−0.07	0.51 *	−0.33 *	0.03	0.02	0.12	0.12	0.20	−0.01	0.73 *	0.51 *	1.00

* Correlations exceeding the threshold of |ρ| ≥ 0.30.

## Data Availability

All data supporting the findings of this study are provided in the Supplementary Materials. Additional details are available from the corresponding author upon reasonable request.

## Supplementary Materials

The following supporting information can be downloaded at: https://www.mdpi.com/article/10.3390/jcm14217631/s1, Table S1: Final dataset (2023–2025): variables, units, coding and data dictionary; Table S2: PCA of the pulmonary profile: eigenvalues, explained variance and component loadings (PC1–PC5); Table S3: Initial pulmonary sub-profiles (P1–P4) and marker composition; Table S4: Refined pulmonary sub-profiles (R1–R11) after repeated PCA: composition and loadings; Table S5: Final profiles and sub-profiles (cardiac, renal, pulmonary): designations and marker sets; Table S6: Cardiac profile reliability: “alpha if item deleted” and Δα for each marker; Table S7: Exploratory factor analysis (EFA): factor structure, loadings (≥0.40), KMO and Bartlett’s test; Table S8: Longitudinal changes (2023–2025) of clinical, functional, laboratory and imaging markers by cohort.

## Abbreviations

The following abbreviations are used in this manuscript:

SSc-ILD	Systemic Sclerosis-associated Interstitial Lung Disease
COPD	Chronic Obstructive Pulmonary Disease
6MWT	Six-Minute Walk Test
6MWD	Six-Minute Walk Distance
SpO_2_	Peripheral Oxygen Saturation
SaO_2_	Arterial Oxygen Saturation
pO_2_	Partial Pressure of Oxygen
pCO_2_	Partial Pressure of Carbon Dioxide
sPAP	Systolic Pulmonary Artery Pressure
LVEF	Left Ventricular Ejection Fraction
NT-proBNP	N-terminal pro–B-type Natriuretic Peptide
MR-proANP	Mid-Regional pro–Atrial Natriuretic Peptide
hsTnT	High-Sensitivity Troponin T
hs-cTnT	High-Sensitivity Cardiac Troponin T
eGFR	Estimated Glomerular Filtration Rate
ACR	Albumin-to-Creatinine Ratio
HRV	Heart Rate Variability
HF	High Frequency (HRV component)
LF	Low Frequency (HRV component)
VLF	Very Low Frequency (HRV component)
TP	Total Power (HRV component)
CT	Computed Tomography
PCA	Principal Component Analysis
FDR	False Discovery Rate
δ (Cliff’s delta)	Cliff’s Delta Effect Size
log_2_FC	Log2 Fold Change
KMO	Kaiser–Meyer–Olkin Test
EFA	Exploratory Factor Analysis
ATS	American Thoracic Society
ASE	American Society of Echocardiography
EACVI	European Association of Cardiovascular Imaging
EULAR	European Alliance of Associations for Rheumatology
GOLD	Global Initiative for Chronic Obstructive Lung Disease

## References

[1] Matthay M. (2014). Resolution of pulmonary edema. Thirty years of progress. Am. J. Respir. Crit. Care Med..

[2] Grams M., Rabb H. (2012). The distant organ effects of acute kidney injury. Kidney Int..

[3] Virzì G., Day S., de Cal M., Vescovo G., Ronco C. (2014). Heart-kidney crosstalk and role of humoral signaling in critical illness. Crit. Care.

[4] Husain-Syed F., McCullough P.A., Birk H.W., Renker M., Brocca A., Seeger W., Ronco C. (2015). Cardio-pulmonary-renal interactions. J. Am. Coll. Cardiol..

[5] Simonneau G., Gatzoulis M.A., Adatia I., Celermajer D., Denton C., Ghofrani A., Sanchez M.A.G., Kumar R.K., Landzberg M., Machado R.F. (2013). Updated clinical classification of pulmonary hypertension. J. Am. Coll. Cardiol..

[6] Azarbar S., Dupuis J. (2014). Lung capillary injury and repair in left heart disease: A new target for therapy?. Clin. Sci..

[7] Hemlin M., Ljungman S., Carlson J., Maljukanovic S., Mobini R., Bech-Hanssen O., Skoogh B. (2007). The effects of hypoxia and hypercapnia on renal and heart function, haemodynamics and plasma hormone levels in stable COPD patients. Clin. Respir. J..

[8] Ronco C., Haapio M., House A.A., Anavekar N., Bellomo R. (2008). Cardiorenal syndrome. J. Am. Coll. Cardiol..

[9] Bollenbecker S., Czaya B., Gutiérrez O.M., Krick S. (2022). Lung–kidney interactions and their role in chronic kidney disease-associated pulmonary diseases. Am. J. Physiol. Lung Cell Mol. Physiol..

[10] Mendes R.S., Silva P.L., Robba C., Battaglini D., Lopes-Pacheco M., Caruso-Neves C., Rocco P.R.M. (2024). Advancements in understanding the mechanisms of lung–kidney crosstalk. Intensive Care Med. Exp..

[11] Takahashi T., Asano Y. (2025). The Evolving Landscape of Systemic Sclerosis Pathogenesis: From Foundational Mechanisms to Organ-Specific Modifiers. Sclerosis.

[12] Park S., Lee S., Kim Y., Cho S., Kim K., Kim Y.C., Han S.S., Lee H., Lee J.P., Joo K.W. (2021). Kidney function and obstructive lung disease: A bidirectional Mendelian randomisation study. Eur. Respir. J..

[13] De Rosa S., Lassola S., Taccone F.S., Battaglini D. (2025). Chronic lung diseases and kidney disease: Pathophysiology and management. Nephrol. Dial. Transplant..

[14] Task Force of the European Society of Cardiology and the North American Society of Pacing and Electrophysiology (1996). Heart rate variability: Standards of measurement, physiological interpretation, and clinical use. Circulation.

[15] (2015). Medical Laboratories—Requirements for Quality and Competence.

[16] Inker L.A., Eneanya N.D., Coresh J., Tighiouart H., Wang D., Sang Y., Crews D.C., Doria A., Estrella M.M., Froissart M. (2021). New creatinine- and cystatin C–based equations to estimate GFR without race. N. Engl. J. Med..

[17] Ibrayeva L., Aubakirova M., Bacheva I., Alina A., Bazarova N., Zhanabayeva A., Avdiyenko O., Borchashvili S., Tazhikhanova S., Murzabaeyev A. (2025). Features of heart failure with preserved ejection fraction in patients with chronic obstructive pulmonary disease and systemic sclerosis-associated interstitial lung diseases. J. Pers. Med..

[18] Ibrayeva L., Bacheva I., Alina A., Klassen O. (2025). Assessing fibrosis progression and endothelial dysfunction in SSc-ILD and COPD: An integrated biomarker and CT densitometry approach. Medicina.

[19] Jurevičienė E., Burneikaitė G., Dambrauskas L., Kasiulevičius V., Kazėnaitė E., Navickas R., Puronaitė R., Smailytė G., Visockienė Ž., Danila E. (2022). Epidemiology of chronic obstructive pulmonary disease (COPD) comorbidities in Lithuanian national database: A cluster analysis. Int. J. Environ. Res. Public Health.

[20] Modak M., Rowlands W.M., Sleiman J., Attaway A.H., Bleecker E.R., Zein J. (2025). Hospitalization outcomes of patients with asthma, COPD, and asthma-COPD overlap syndrome. Chronic Obstr. Pulm. Dis..

[21] Kvisvik B., Skjørten I., Hilde J.M., Strand H., Omland T., Steine K. (2019). High-sensitivity troponin T predicts mortality independently of ventricular dysfunction and pulmonary hypertension in stable chronic obstructive pulmonary disease. Circulation.

[22] Sá-Sousa A., Rodrigues C., Jácome C., Cardoso J., Fortuna I., Guimarães M., Pinto P., Sarmento P.M., Baptista R. (2024). Cardiovascular risk in patients with chronic obstructive pulmonary disease: A systematic review. J. Clin. Med..

[23] Knarborg M., Hyldgaard C., Bendstrup E., Davidsen J.R., Løkke A., Shaker S.B., Hilberg O. (2023). Comorbidity and mortality in systemic sclerosis and matched controls: Impact of interstitial lung disease. Chron. Respir. Dis..

[24] Su X., Lei T., Yu H., Zhang L., Feng Z., Shuai T., Guo H., Liu J. (2023). NT-proBNP in different patient groups of COPD: A systematic review and meta-analysis. Int. J. Chronic Obstr. Pulm. Dis..

[25] Ma K.K., Ogawa T., de Bold A.J. (2004). Selective upregulation of cardiac brain natriuretic peptide at the transcriptional and translational levels by pro-inflammatory cytokines and by conditioned medium derived from mixed lymphocyte reactions via p38 MAP kinase. J. Mol. Cell Cardiol..

[26] Liu Z., Ma Z., Ding C. (2024). Association between COPD and CKD: A systematic review and meta-analysis. Front. Public Health.

[27] Konstantinidou S.K., Argyrakopoulou G., Tentolouris N., Karalis V., Kokkinos A. (2022). Interplay between baroreflex sensitivity, obesity and related cardiometabolic risk factors (Review). Exp. Ther. Med..

[28] Kabbach E.Z., Mazzuco A., Borghi-Silva A., Cabiddu R., Agnoleto A.G., Barbosa J.F., Junior L.C.S.d.C., Mendes R.G. (2017). Increased parasympathetic cardiac modulation in patients with acute exacerbation of COPD: How should we interpret it?. Int. J. Chronic Obstr. Pulm. Dis..

[29] Guo P., Li R., Piao T.H., Wang C.L., Wu X.L., Cai H.Y. (2022). Pathological mechanism and targeted drugs of COPD. Int. J. Chronic Obstr. Pulm. Dis..

[30] Gao J., Wang A., Li X., Li J., Zhao H., Zhang J., Liang J., Chen S., Wu S. (2020). The cumulative exposure to high-sensitivity C-reactive protein predicts the risk of chronic kidney diseases. Kidney Blood Press. Res..

[31] Elmahallawy I.I., Qora M.A. (2013). Prevalence of chronic renal failure in COPD patients. Egypt. J. Chest Dis. Tuberc..

[32] Vilstrup F., Heerfordt C.K., Kamstrup P., Hedsund C., Biering-Sørensen T., Sørensen R., Kolekar S., Hilberg O., Pedersen L., Lund T.K. (2023). Renin-angiotensin-system inhibitors and the risk of exacerbations in chronic obstructive pulmonary disease: A nationwide registry study. BMJ Open Respir. Res..

