# Supplementary material for: Pulmo–Cardio–Renal Continuum in Chronic Lung Diseases: A 3-Year Prospective Cohort Study
Source: J Clin Med. 2025 Oct 28;14(21):7631. doi: 10.3390/jcm14217631 (PMC12608822; doi:10.3390/jcm14217631)
Supplement: Supplementary file 1 [file jcm-14-07631-s001.zip › Supplementary 8.pdf]

Variables	Year	ME [Q1;Q3] (ILD-SSD) (n=59)	P (ILD-SSD)*	q-value (ILD-SSD)*	ME [Q1;Q3] COPD	P (COPD) (n=62)*	q-value (COPD)*	p_ ((ILD-SSD) - COPD)**	q-value**
SBP before 6MWT	2023	120 [110; 120]			120 [110; 125]			0,18	0,29
	2024	115 [110; 120]	0,28	0,37	120 [110; 120]	0,29	0,37	0,33	0,49
	2025	110 [110; 120]	0,02	0,03	120 [110; 129]	0,00	0,00	0,09	0,15
SBP after 6MWT	2023	120 [120; 130]			125 [120; 130]			0,88	0,90
	2024	120 [120; 130]	0,51	0,56	125 [120; 130]	0,14	0,19	0,57	0,65
	2025	130 [120; 140]	0,00	0,00	130 [120; 140]	0,00	0,00	0,36	0,49
DBP before 6MWT	2023	70 [70; 80]			80 [70; 80]			0,04	0,07
	2024	70 [70; 80]	0,62	0,67	70 [70; 80]	0,01	0,02	0,93	0,93
	2025	70 [70; 80]	0,20	0,26	80 [70; 80]	0,01	0,01	0,42	0,54
DBP after 6MWT	2023	80 [80; 90]			80 [80; 90]			0,81	0,84
	2024	80 [80; 90]	0,99	0,99	80 [80; 90]	0,35	0,43	0,54	0,63
	2025	80 [80; 90]	0,00	0,00	90 [80; 90]	0,00	0,00	0,36	0,49
SaO_2 before 6MWT	2023	98 [95.5; 98]			95.5 [94; 96]			0,00	0,00
	2024	97 [96; 98]	0,01	0,02	95 [93.2; 96]	0,14	0,20	0,00	0,00
	2025	97 [95; 97]	0,00	0,00	95 [92; 96]	0,00	0,00	0,00	0,00
SaO_2 after 6MWT	2023	95 [93.5; 97]			89.5 [88; 94.8]			0,00	0,00
	2024	96 [92.5; 96]	0,07	0,11	89 [88; 93]	0,02	0,03	0,00	0,00
	2025	95 [90; 95]	0,00	0,00	87.5 [85.2; 91]	0,00	0,00	0,00	0,00
6MWT Distance	2023	300 [235; 358]			245 [180; 320]			0,02	0,04
	2024	300 [230; 350]	0,33	0,42	245 [180; 315]	0,05	0,07	0,01	0,02
	2025	280 [220; 335]	0,00	0,00	235 [172; 300]	0,00	0,00	0,00	0,01
HR before 6MWT	2023	76 [70; 80]			81.5 [76.2; 87]			0,00	0,00
	2024	78 [72; 82]	0,93	0,95	83.5 [76; 90]	0,42	0,49	0,00	0,01
	2025	80 [76; 85]	0,00	0,00	86 [78.5; 91.5]	0,00	0,00	0,00	0,00
HR after 6MWT	2023	89 [84; 94]			94 [85; 102]			0,06	0,11
	2024	88 [84; 94.5]	0,52	0,56	95 [85; 103]	0,50	0,56	0,03	0,05
	2025	90 [88; 96]	0,00	0,00	98 [90.5; 106]	0,00	0,00	0,00	0,01
RR before 6MWT	2023	19 [17.5; 20]			20 [19; 20.8]			0,00	0,00
	2024	18 [18; 20]	0,89	0,93	20 [19; 20]	0,85	0,90	0,00	0,00

	2025	19 [19; 21]	0,00	0,00	20 [20; 22]	0,00	0,00	0,00	0,01
RR after 6MWT	2023	22 [20; 26]			24 [22; 26]			0,04	0,07
	2024	22 [20; 24.5]	0,37	0,45	24 [23.2; 26]	0,10	0,15	0,00	0,00
	2025	23 [22; 26.5]	0,00	0,00	25 [24; 27]	0,00	0,00	0,00	0,00
ΔRR 6MWT	2023	3 [2; 4.5]			4 [2; 6]			0,63	0,71
	2024	3 [2; 4]	0,38	0,45	4 [4; 5]	0,04	0,06	0,00	0,01
	2025	4 [3; 5]	0,00	0,00	5 [4; 6]	0,00	0,00	0,01	0,01
Borg scale before 6MWT	2023	1 [0.5; 2]			1 [1; 2]			0,20	0,32
	2024	1 [1; 2]	0,48	0,55	2 [1; 2]	0,34	0,42	0,01	0,03
	2025	1 [1; 2]	0,00	0,00	2 [1; 3]	0,00	0,00	0,00	0,01
Borg scale after 6MWT	2023	3 [1; 4]			3 [3; 5]			0,02	0,04
	2024	3 [2; 4]	0,96	0,97	4 [3; 5]	0,01	0,02	0,00	0,00
	2025	3 [2; 4]	0,00	0,00	5 [4; 6]	0,00	0,00	0,00	0,00
Lac	2023	1.21 [0.905; 1.7]			1.4 [1.08; 2]			0,10	0,18
	2024	1.25 [0.85; 1.9]	0,05	0,08	1.38 [1.02; 2.37]	0,02	0,03	0,15	0,26
	2025	1.4 [1.08; 2.06]	0,00	0,00	1.54 [1.24; 2.47]	0,00	0,00	0,26	0,40
pCO2	2023	40 [37.5; 45.6]			41.8 [36; 48.2] (n=60)			0,70	0,77
	2024	41.5 [38.2; 46.7]	0,00	0,00	43.8 [36.7; 50.3] (n=60)	0,00	0,00	0,37	0,49
	2025	42.8 [40.3; 48.4]	0,00	0,00	44.8 [39.5; 50.4] (n=60)	0,00	0,00	0,36	0,49
pH	2023	7.4 [7.38; 7.44]			7.41 [7.36; 7.43]			0,35	0,49
	2024	7.43 [7.4; 7.45]	0,00	0,00	7.43 [7.38; 7.45] (n=60)	0,00	0,00	0,78	0,84
	2025	7.43 [7.41; 7.46]	0,00	0,00	7.44 [7.39; 7.46] (n=60)	0,00	0,00	0,79	0,84
pO2	2023	78.7 [72.5; 84.5]			80.5 [73; 88.2] (n=60)			0,51	0,61
	2024	73.6 [70; 82.7]	0,00	0,00	78.3 [69; 86.4] (n=60)	0,00	0,00	0,45	0,55
	2025	72.5 [67.5; 80]	0,00	0,00	76.7 [68.3; 84.1] (n=60)	0,00	0,00	0,44	0,55
Exhaled CO_2	2023	30 [27,2; 36]			28,5 [22; 34]			0,0489	0,0799

	2024	28 [25,2; 34,8]	0,0000	0,0000	27,5 [20; 31,8] (n=62)	0,0000	0,0000	0,18	0,25
	2025	27,5 [25; 34,2]	0,001	0,003	27 [20; 31] (n=62)	0,21	0,26	0,23	0,3
FVC1	2023	74 [84,7; 60,4]			45,8 [64,5; 31,6]			0,0000	0,0000
	2024	76,5 [87,4; 63,4]	0,08	0,11	40,5 [58,1; 30,8]	0,39	0,44	0,0000	0,0000
	2025	75,2 [82,3; 60,4]	0,0000	0,0000	38 [55,4; 30,2]	0,0000	0,0000	0,0000	0,0000
FEV	2023	-72 [-89,6; -59,5] (n=59)			-71,2 [-95; -54,9] (n=59)			1,0000	1,0000
	2024	-72,8 [-92,9; -58,5] (n=59)	0,78	0,82	-68,8 [-89; -48,5]	0,0052	0,0092	0,1442	0,2163
	2025	-71,5 [-87,5; -55,2] (n=59)	0,0000	0,0000	-65,5 [-84,9; -45,8]	0,0000	0,0000	0,1929	0,2671
Lung volume	2023	3360 [4020; 2740]			6750 [7670; 5930]			0,0000	0,0000
	2024	3250 [3830; 2550]	0,005	0,009	6990 [7510; 5740]	0,04	0,05	0,0000	0,0000
	2025	3120 [3610; 2370]	0,0001	0,0002	6890 [7480; 5610]	0,41	0,44	0,0000	0,0000
Densitometry parameters	2023	-779 [-829; -742]			-884 [-911; -860]			0,0000	0,0000
	2024	-790 [-831; -720]	0,99	0,99	-882 [-914; -858]	0,51	0,54	0,0000	0,0000
	2025	-788 [-828; -712]	0,14	0,19	-882 [-915; -854]	0,87	0,89	0,0000	0,0000
Fibrosis density	2023	-483 [-606; -322]			-475 [-620; -385]			0,27	0,34
	2024	-443 [-589; -324]	0,53	0,56	-486 [-620; -412]	0,003	0,006	0,07	0,12
	2025	-435 [-578; -320]	0,002	0,003	-492 [-624; -420]	0,37	0,44	0,04	0,06

Intergroup p (Mann–Whitney) + q_fdr_bh usually denotes a p-value adjusted by the Benjamini–Hochberg (BH) method to control the False Discovery Rate (FDR).

- **q_fdr_bh < 0.05** → the result is statistically significant after correction for multiple comparisons.
- The smaller the **q_fdr_bh**, the more reliable the statistical significance.

* **Intragroup p (Wilcoxon, only adjacent years: 2024 vs 2023 and 2025 vs 2024) + FDR (BH)**

Variables	Years	Delta ME, IQR (ILD-SSD) (n=59)	P (ILD-SSD)*	q-value (ILD-SSD)*	Delta ME, IQR COPD	P (COPD) (n=62)*	q-value (COPD)*	p_ ((ILD-SSD) - COPD)**	q-value**
SBP before 6MWT	Δ(2024–2023)	120 [110; 120]			120 [110; 125]			0,18	0,29
	Δ(2025–2024)	115 [110; 120]	0,28	0,37	120 [110; 120]	0,29	0,37	0,33	0,49
	Δ(2025–2023)	110 [110; 120]	0,02	0,03	120 [110; 129]	0,00	0,00	0,09	0,15
SBP after 6MWT	Δ(2024–2023)	120 [120; 130]			125 [120; 130]			0,88	0,90
	Δ(2025–2024)	120 [120; 130]	0,51	0,56	125 [120; 130]	0,14	0,19	0,57	0,65
	Δ(2025–2023)	130 [120; 140]	0,00	0,00	130 [120; 140]	0,00	0,00	0,36	0,49
DBP before 6MWT	Δ(2024–2023)	70 [70; 80]			80 [70; 80]			0,04	0,07
	Δ(2025–2024)	70 [70; 80]	0,62	0,67	70 [70; 80]	0,01	0,02	0,93	0,93
	Δ(2025–2023)	70 [70; 80]	0,20	0,26	80 [70; 80]	0,01	0,01	0,42	0,54
DBP after 6MWT	Δ(2024–2023)	80 [80; 90]			80 [80; 90]			0,81	0,84
	Δ(2025–2024)	80 [80; 90]	0,99	0,99	80 [80; 90]	0,35	0,43	0,54	0,63
	Δ(2025–2023)	80 [80; 90]	0,00	0,00	90 [80; 90]	0,00	0,00	0,36	0,49
SaO_2 before 6MWT	Δ(2024–2023)	98 [95.5; 98]			95.5 [94; 96]			0,00	0,00
	Δ(2025–2024)	97 [96; 98]	0,01	0,02	95 [93.2; 96]	0,14	0,20	0,00	0,00
	Δ(2025–2023)	97 [95; 97]	0,00	0,00	95 [92; 96]	0,00	0,00	0,00	0,00
SaO_2 after 6MWT	Δ(2024–2023)	95 [93.5; 97]			89.5 [88; 94.8]			0,00	0,00
	Δ(2025–2024)	96 [92.5; 96]	0,07	0,11	89 [88; 93]	0,02	0,03	0,00	0,00
	Δ(2025–2023)	95 [90; 95]	0,00	0,00	87.5 [85.2; 91]	0,00	0,00	0,00	0,00
6MWT Distance	Δ(2024–2023)	300 [235; 358]			245 [180; 320]			0,02	0,04
	Δ(2025–2024)	300 [230; 350]	0,33	0,42	245 [180; 315]	0,05	0,07	0,01	0,02
	Δ(2025–2023)	280 [220; 335]	0,00	0,00	235 [172; 300]	0,00	0,00	0,00	0,01
HR before 6MWT	Δ(2024–2023)	76 [70; 80]			81.5 [76.2; 87]			0,00	0,00
	Δ(2025–2024)	78 [72; 82]	0,93	0,95	83.5 [76; 90]	0,42	0,49	0,00	0,01
	Δ(2025–2023)	80 [76; 85]	0,00	0,00	86 [78.5; 91.5]	0,00	0,00	0,00	0,00
HR after 6MWT	Δ(2024–2023)	89 [84; 94]			94 [85; 102]			0,06	0,11
	Δ(2025–2024)	88 [84; 94.5]	0,52	0,56	95 [85; 103]	0,50	0,56	0,03	0,05
	Δ(2025–2023)	90 [88; 96]	0,00	0,00	98 [90.5; 106]	0,00	0,00	0,00	0,01
RR before 6MWT	Δ(2024–2023)	19 [17.5; 20]			20 [19; 20.8]			0,00	0,00
	Δ(2025–2024)	18 [18; 20]	0,89	0,93	20 [19; 20]	0,85	0,90	0,00	0,00

	Δ(2025–2023)	19 [19; 21]	0,00	0,00	20 [20; 22]	0,00	0,00	0,00	0,01
RR after 6MWT	Δ(2024–2023)	22 [20; 26]			24 [22; 26]			0,04	0,07
	Δ(2025–2024)	22 [20; 24.5]	0,37	0,45	24 [23.2; 26]	0,10	0,15	0,00	0,00
	Δ(2025–2023)	23 [22; 26.5]	0,00	0,00	25 [24; 27]	0,00	0,00	0,00	0,00
ΔRR 6MWT	Δ(2024–2023)	3 [2; 4.5]			4 [2; 6]			0,63	0,71
	Δ(2025–2024)	3 [2; 4]	0,38	0,45	4 [4; 5]	0,04	0,06	0,00	0,01
	Δ(2025–2023)	4 [3; 5]	0,00	0,00	5 [4; 6]	0,00	0,00	0,01	0,01
Borg scale before 6MWT	Δ(2024–2023)	1 [0.5; 2]			1 [1; 2]			0,20	0,32
	Δ(2025–2024)	1 [1; 2]	0,48	0,55	2 [1; 2]	0,34	0,42	0,01	0,03
	Δ(2025–2023)	1 [1; 2]	0,00	0,00	2 [1; 3]	0,00	0,00	0,00	0,01
Borg scale after 6MWT	Δ(2024–2023)	3 [1; 4]			3 [3; 5]			0,02	0,04
	Δ(2025–2024)	3 [2; 4]	0,96	0,97	4 [3; 5]	0,01	0,02	0,00	0,00
	Δ(2025–2023)	3 [2; 4]	0,00	0,00	5 [4; 6]	0,00	0,00	0,00	0,00
Lac	Δ(2024–2023)	0,13; 0,48	0,029*		0.13; 0.48	0,018*	0,029*	0,10	0,18
	Δ(2025–2024)	0,16 [0,1;0,5425]	<0,0001*	0,08	0.14;0.11]	<0,0001*	<0,0001*	0,15	0,26
	Δ(2025–2023)	0.28	<0,0001*	0,00	0,355 [0,072;0,503]	<0,0001*	<0,0001*	0,26	0,40
pCO2	Δ(2024–2023)	40 [37.5; 45.6]			41.8 [36; 48.2] (n=60)			0,70	0,77
	Δ(2025–2024)	41.5 [38.2; 46.7]	0,00	0,00	43.8 [36.7; 50.3] (n=60)	0,00	0,00	0,37	0,49
	Δ(2025–2023)	42.8 [40.3; 48.4]	0,00	0,00	44.8 [39.5; 50.4] (n=60)	0,00	0,00	0,36	0,49
pH	Δ(2024–2023)	7.4 [7.38; 7.44]			7.41 [7.36; 7.43]			0,35	0,49
	Δ(2025–2024)	7.43 [7.4; 7.45]	0,00	0,00	7.43 [7.38; 7.45] (n=60)	0,00	0,00	0,78	0,84
	Δ(2025–2023)	7.43 [7.41; 7.46]	0,00	0,00	7.44 [7.39; 7.46] (n=60)	0,00	0,00	0,79	0,84
pO2	Δ(2024–2023)	78.7 [72.5; 84.5]			80.5 [73; 88.2] (n=60)			0,51	0,61
	Δ(2025–2024)	73.6 [70; 82.7]	0,00	0,00	78.3 [69; 86.4] (n=60)	0,00	0,00	0,45	0,55
	Δ(2025–2023)	72.5 [67.5; 80]	0,00	0,00	76.7 [68.3; 84.1] (n=60)	0,00	0,00	0,44	0,55
Exhaled CO_2	Δ(2024–2023)	30 [27,2; 36]			28,5 [22; 34]			0,0489	0,0799

	Δ(2025–2024)	28 [25,2; 34,8]	0,0000	0,0000	27,5 [20; 31,8] (n=62)	0,0000	0,0000	0,18	0,25
	Δ(2025–2023)	27,5 [25; 34,2]	0,001	0,003	27 [20; 31] (n=62)	0,21	0,26	0,23	0,3
FVC1	Δ(2024–2023)	74 [84,7; 60,4]			45,8 [64,5; 31,6]			0,0000	0,0000
	Δ(2025–2024)	76,5 [87,4; 63,4]	0,08	0,11	40,5 [58,1; 30,8]	0,39	0,44	0,0000	0,0000
	Δ(2025–2023)	75,2 [82,3; 60,4]	0,0000	0,0000	38 [55,4; 30,2]	0,0000	0,0000	0,0000	0,0000
FEV	Δ(2024–2023)	-72 [-89,6; -59,5] (n=59)			-71,2 [-95; -54,9] (n=59)			1,0000	1,0000
	Δ(2025–2024)	-72,8 [-92,9; -58,5] (n=59)	0,78	0,82	-68,8 [-89; -48,5]	0,0052	0,0092	0,1442	0,2163
	Δ(2025–2023)	-71,5 [-87,5; -55,2] (n=59)	0,0000	0,0000	-65,5 [-84,9; -45,8]	0,0000	0,0000	0,1929	0,2671
Lung volume	Δ(2024–2023)	3360 [4020; 2740]			6750 [7670; 5930]			0,0000	0,0000
	Δ(2025–2024)	3250 [3830; 2550]	0,005	0,009	6990 [7510; 5740]	0,04	0,05	0,0000	0,0000
	Δ(2025–2023)	3120 [3610; 2370]	0,0001	0,0002	6890 [7480; 5610]	0,41	0,44	0,0000	0,0000
Densitometry parameters	Δ(2024–2023)	-779 [-829; -742]			-884 [-911; -860]			0,0000	0,0000
	Δ(2025–2024)	-790 [-831; -720]	0,99	0,99	-882 [-914; -858]	0,51	0,54	0,0000	0,0000
	Δ(2025–2023)	-788 [-828; -712]	0,14	0,19	-882 [-915; -854]	0,87	0,89	0,0000	0,0000
Fibrosis density	Δ(2024–2023)	-483 [-606; -322]			-475 [-620; -385]			0,27	0,34
	Δ(2025–2024)	-443 [-589; -324]	0,53	0,56	-486 [-620; -412]	0,003	0,006	0,07	0,12
	Δ(2025–2023)	-435 [-578; -320]	0,002	0,003	-492 [-624; -420]	0,37	0,44	0,04	0,06

Variables	Year	ME [Q1;Q3] (ILD-SSD) (n=59)	P (ILD-SSD)*	q-value (ILD-SSD)*	ME [Q1;Q3] COPD	P (COPD) (n=62)*	q-value (COPD)*	p_ ((ILD-SSD) - COPD)**	q-value**
HRC IN	2023	28.6 [14.5; 40.0]			24.4 [10.8; 38.8]			0,557	0,619
	2024	16.3 [10.4; 29.95]	0,003	0,032	19.55 [10.33; 32.38]	0,458	0,508	0,547	0,684
	2025	4.0 [-1.9; 17.65]	0	0	14.7 [5.475; 27.525]	0,009	0,012	0	0,003
HRC HF	2023	34.2 [25.1; 45.1]			39.45 [24.68; 51.4]			0,353	0,505
	2024	38.4 [34.85; 49.7]	0,015	0,055	46.55 [29.7; 54.9]	0,223	0,382	0,251	0,358
	2025	42.6 [39.05; 53.9]	0	0	53.65 [36.8; 62.0]	0	0	0,038	0,063
HRC LF	2023	620.0 [426.0; 1267.0]			759.5 [359.0; 1674.75]			0,851	0,851
	2024	509.0 [285.0; 813.0]	0,255	0,382	515.5 [229.0; 1296.75]	0,268	0,382	0,694	0,771
	2025	398.0 [174.0; 702.0]	0,007	0,011	271.5 [-15.0; 1052.75	0,004	0,006	0,224	0,249
HRC VLF	2023	120 [120; 130]			125 [120; 130]			0,88	0,90
	2024	120 [120; 130]	0,51	0,56	125 [120; 130]	0,14	0,19	0,57	0,65
	2025	130 [120; 140]	0,00	0,00	130 [120; 140]	0,00	0,00	0,36	0,49
HRC TP	2023	70 [70; 80]			80 [70; 80]			0,04	0,07
	2024	70 [70; 80]	0,62	0,67	70 [70; 80]	0,01	0,02	0,93	0,93
	2025	70 [70; 80]	0,20	0,26	80 [70; 80]	0,01	0,01	0,42	0,54
ntproBNP	2023	120 [120; 130]			125 [120; 130]			0,88	0,90
	2024	120 [120; 130]	0,51	0,56	125 [120; 130]	0,14	0,19	0,57	0,65
	2025	130 [120; 140]	0,00	0,00	130 [120; 140]	0,00	0,00	0,36	0,49
MR-proANP	2023	70 [70; 80]			80 [70; 80]			0,04	0,07
	2024	70 [70; 80]	0,62	0,67	70 [70; 80]	0,01	0,02	0,93	0,93
	2025	70 [70; 80]	0,20	0,26	80 [70; 80]	0,01	0,01	0,42	0,54
hsTnT	2023	120 [120; 130]			125 [120; 130]			0,88	0,90
	2024	120 [120; 130]	0,51	0,56	125 [120; 130]	0,14	0,19	0,57	0,65
	2025	130 [120; 140]	0,00	0,00	130 [120; 140]	0,00	0,00	0,36	0,49
LVEF	2023	70 [70; 80]			80 [70; 80]			0,04	0,07
	2024	70 [70; 80]	0,62	0,67	70 [70; 80]	0,01	0,02	0,93	0,93
	2025	70 [70; 80]	0,20	0,26	80 [70; 80]	0,01	0,01	0,42	0,54
sPAP	2023	70 [70; 80]			80 [70; 80]			0,04	0,07

	2024	70 [70; 80]	0,62	0,67	70 [70; 80]	0,01	0,02	0,93	0,93
	2025	70 [70; 80]	0,20	0,26	80 [70; 80]	0,01	0,01	0,42	0,54

Marker	Year	ME [Q1;Q3] (ILD-SSD) (n)	P (ILD-SSD)*	q-value (ILD-SSD)*	ME [Q1;Q3] COPD(n)	P (COPD)*	q-value (COPD)*	p_ ((ILD-SSD) - COPD)**	q-value**
hrv_tp	2023	(n=55)			(n=62)				
hrv_tp	2024	(n=59)			(n=62)				
hrv_tp	2025	(n=59)			] (n=62)				
hrv_vlf	2023	34.7 [26.4; 48.5] (n=57)			31.35 [18.2; 42.275] (n=62)			0,209	0,349
hrv_vlf	2024	38.6 [28.85; 49.1] (n=59)	0,854	0,854	31.05 [19.825; 42.05] (n=62)	0,662	0,662	0,026	0,132
hrv_vlf	2025	42.5 [32.75; 53.0] (n=59)	0,084	0,105	30.75 [19.525; 41.75] (n=62)	0,75	0,75	0,001	0,004
hrv_ин	2023	373.0 [178.0; 611.0] (n=57)			454.0 [197.5; 902.75] (n=62)			0,437	0,547
hrv_ин	2024	511.0 [302.0; 911.0] (n=59)	0,098	0,196	652.5 [339.25; 1393.0] (n=62)	0,051	0,17	0,175	0,293
hrv_ин	2025	649.0 [440.0; 1049.0] (n=59)	0	0	851.0 [537.75; 1591.5] (n=62)	0	0	0,035	0,063
hs_tnt	2023	1.224 [0.82; 2.129] (n=59)			1.071 [0.617; 1.529] (n=62)			0,138	0,276
hs_tnt	2024	1.303 [0.904; 1.749] (n=59)	0,618	0,773	1.448 [0.868; 1.852] (n=62)	0,011	0,054	0,915	0,915
hs_tnt	2025	1.358 [0.974; 1.693] (n=58)	0,528	0,528	1.37 [0.956; 1.983] (n=62)	0,001	0,002	0,815	0,815
lvef	2023	65.0 [61.0; 67.0] (n=59)			62.0 [58.0; 66.0] (n=62)			0,009	0,03
lvef	2024	63.0 [59.5; 66.0] (n=59)	0,045	0,113	61.0 [57.25; 65.0] (n=62)	0,318	0,398	0,058	0,192
lvef	2025	60.0 [54.0; 62.0] (n=59)	0	0	58.0 [54.25; 60.0] (n=62)	0	0	0,163	0,204
mr_proanp	2023	31.246 [14.529; 77.76] (n=59)			18.073 [6.044; 32.237] (n=62)			0,005	0,025
mr_proanp	2024	49.378 [26.453; 67.783] (n=59)	0,267	0,382	30.445 [13.191; 45.863] (n=62)	0	0,001	0,004	0,035
mr_proanp	2025	64.755 [44.116; 84.556] (n=59)	0,01	0,014	45.65 [21.268; 61.742] (n=62)	0	0	0,003	0,009
ntprobnp	2023	93.62 [34.845; 218.1] (n=59)			58.05 [28.095; 134.225] (n=62)			0,101	0,253
ntprobnp	2024	106.6 [46.85; 204.8] (n=59)	0,717	0,797	78.5 [47.4; 119.35] (n=62)	0,186	0,382	0,098	0,245

ntprobnp	2025	128.6 [75.6; 196.482] (n=59)	0,216	0,24	97.4 [58.775; 135.6] (n=62)	0,024	0,026	0,079	0,113
pap	2023	20.0 [12.5; 21.5] (n=59)			20.0 [20.0; 30.0] (n=62)			0,005	0,025
pap	2024	20.0 [20.0; 28.0] (n=59)	0,017	0,055	25.0 [20.0; 30.0] (n=62)	0,258	0,382	0,176	0,293
pap	2025	25.0 [23.0; 32.5] (n=59)	0	0	30.0 [25.0; 35.75] (n=62)	0	0	0,007	0,017
